# Self‐Assembling Multi‐Antigen T Cell Hybridizers for Precision Immunotherapy of Multiple Myeloma

**DOI:** 10.1002/adhm.202502156

**Published:** 2025-08-01

**Authors:** Shannuo Li, Jiahui Li, Hasan Al Faruque, Paul Shami, Birgit Knoechel, Jens Lohr, Douglas Sborov, Jiyuan Yang, Jindřich Kopeček

**Affiliations:** ^1^ Department of Molecular Pharmaceutics University of Utah Salt Lake City UT 84112 USA; ^2^ Center for Controlled Chemical Delivery University of Utah Salt Lake City UT 84112 USA; ^3^ Huntsman Cancer Institute University of Utah Salt Lake City UT 84112 USA; ^4^ Department of Biomedical Engineering University of Utah Salt Lake City UT 84112 USA

**Keywords:** B‐cell maturation antigen (BCMA), drug‐free macromolecular therapeutics, morpholino oligonucleotides, multiple myeloma, T cell‐redirected immunotherapy

## Abstract

Bispecific T‐cell engagers show promise in treating multiple myeloma (MM), but challenges remain in adaptability and targeting flexibility. This paper presents a novel T‐cell based immunotherapy, Multi‐Antigen TCell Hybridizers (MATCH), a modular, self‐assembling T‐cell engager designed for versatile and patient‐specific cancer targeting. MATCH consists of two components: a B‐cell‐targeting Fab’ fragment conjugated to a 25‐base morpholino oligonucleotide (Fab’_B cell antigen_‐MORF1) and a T‐cell engaging anti‐CD3 Fab’ fragment conjugated to the complementary morpholino oligonucleotide (Fab’_CD3_‐MORF2). Upon hybridization of MORF1 and MORF2, MATCH enables pre‐targeting of malignant cells followed by in situ post‐assembly of the bispecific complex, facilitating targeted T‐cell recruitment. To enhance antigen specificity based on MM patient expression profile, a panel of Fab’‐MORF1 conjugates targeting key MM surface markers (Fab’_BCMA_‐MORF1, Fab’_SLAMF7_‐MORF1, Fab’_CD38_‐MORF1) is developed, which pairs interchangeably with Fab’_CD3_‐MORF2 for T‐cell engagement. MATCH effectively induces immune synapse formation and exhibits potent, antigen‐specific cytotoxicity across MM cells. Ex vivo validation in patient‐derived bone marrow samples confirms significant tumor cell depletion. Preliminary in vivo studies in humanized mouse model demonstrated effective cancer inhibition along with favorable pharmacokinetics and distribution profiles. These findings support MATCH as a flexible and customizable immunotherapy platform with strong translational potential for the treatment of MM.

## Graphical abstract

A novel modular T‐cell engager platform, MATCH, is developed for personalized multiple myeloma therapy. MATCH uses complementary oligonucleotide‐linked Fab’ fragments to enable flexible targeting of MM antigens and controlled T‐cell activation. The system shows potent, antigen‐specific cytotoxicity in vitro and in vivo, highlighting its potential for adaptable, patient‐specific immunotherapy.

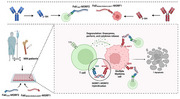

## 1 Introduction

Multiple myeloma (MM) is a hematologic malignancy characterized by the uncontrolled proliferation of plasma cells in the bone marrow, accounting for ≈13% of all blood cancers in the United States. Despite traditional therapeutic approaches, such as chemotherapy, monoclonal antibodies, immunomodulatory agents, proteosome inhibitors, and autologous stem cell transplantation that have extended patient survival over the past two decades, MM remains incurable in part due to the heterogeneity of MM cell populations with frequent relapses and eventual progression to refractory disease.^[^ ^1^, ^2^ ^]^ The advent of immunotherapies has offered a promising new avenue for MM treatment, which harnesses patients own immune system to target and eliminate cancer cells. Notably, chimeric antigen receptor (CAR) T cell therapies and T‐cell redirecting bispecific antibodies against B‐cell maturation antigen (BCMA) and G‐protein coupled receptor Class C Group 5D (GPRC5D), have demonstrated encouraging efficacy by harnessing cytotoxic T cells to selectively eliminate MM cells.^[^ ^3^, ^4^, ^5^, ^6^ ^]^ For example, teclistamab and elranatamab are bispecifics that target BCMA on MM cells and CD3 on T cells, create an immunological synapse that triggers T‐cell activation, and ultimately kill MM cells.^[^ ^7^, ^8^ ^]^ These drugs, and others, represent a huge step forward in providing off‐the‐shelf therapeutics that prolong patient's lives.

However, bispecifics are limited by their fixed antigen specificity, where antigen expression may be variable and diminish under therapeutic pressure, potentially leading to the emergence of negative or low‐expression clones of specific targets that can escape immune surveillance.^[^ ^9^ ^]^ Recent efforts to overcome these limitations have focused on developing multi‐specific T‐cell recruiting strategies capable of targeting multiple antigens simultaneously, such as multi‐specific T‐cell engagers, multi‐antigen CAR‐T cells.^[^ ^10^, ^11^ ^]^ Multi‐targeting approaches may potentially prevent relapse due to antigen downregulation or heterogeneity. But still, the fixed antigen specificity makes them difficult to adapt to changing antigen profiles, which can be costly and time‐consuming to develop new targets for individual patients.

**Figure 1:**
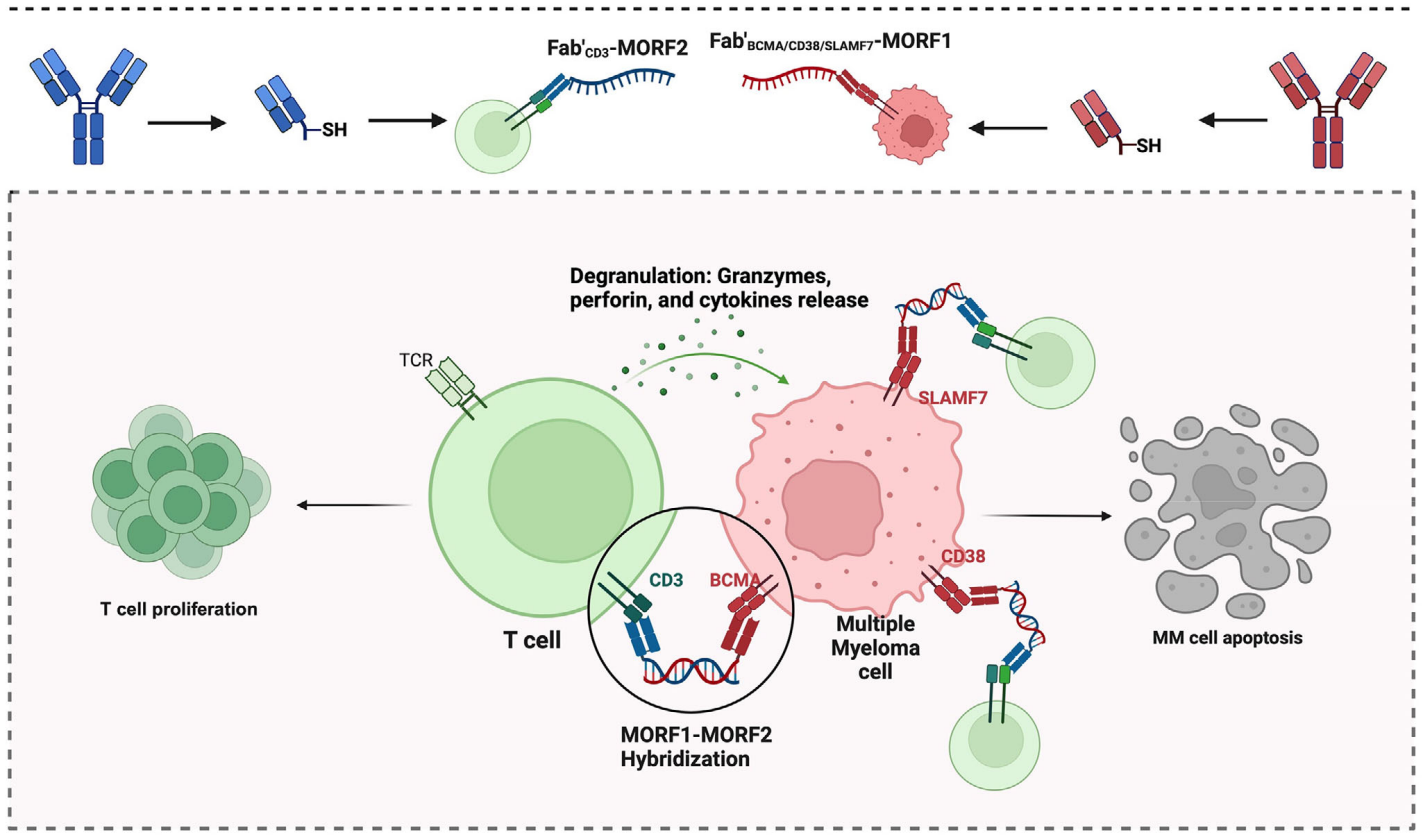
Schematic illustration of the design and proposed mechanism of action of the Multi‐Antigen T Cell Hybridizer (MATCH) platform for targeted T cell‐mediated killing of multiple myeloma (MM) cells. The construction of the MATCH complex starts with whole antibodies, which are digested and reduced to Fab’ fragments, that are then conjugated to a 25‐base pair morpholino oligonucleotide (MORF), allowing for specific self‐assembly of Fab’_BCMA/CD38/SLAMF7_‐MORF1 and Fab’_CD3_‐MORF2 in vitro and in vivo. Upon MORF1–MORF2 hybridization, the assembled MATCH complex brings T cells into close proximity with MM cells, promoting immune synapse formation and targeted cytotoxicity. This multi‐antigen approach enhances tumor cell killing through both direct cytolysis and apoptosis signaling. Figure created with BioRender.com.

In this study, we introduce the Multi‐Antigen TCell Hybridizer (MATCH), a novel and flexible T‐cell engagement platform designed to address the limitations of current T‐cell therapies for MM. MATCH is a modular, self‐assembling two‐component system that employs antibody Fab’ fragments conjugated to 25‐base complementary morpholino oligonucleotide (MORF) sequences.^[^ ^12^, ^13^ ^]^ This design allows Fab’ fragments to bind specific antigens on MM cells and T cells respectively, while the complementary MORFs self‐assemble and bridge T cells toward MM cells by hybridization, forming immune synapses (**Figure** **1**). Unlike traditional bispecifics, MATCH enables customizability in antigen targeting by allowing the flexible selection of MM antigen‐specific Fab’ fragments from a minibank of Fab’ conjugates (Fab’_BCMA_‐MORF1, Fab’_SLAMF7_‐MORF1, Fab’_CD38_‐MORF1) in combination with a T cell‐engaging Fab’_CD3_‐MORF2 component. This split‐antibody design not only enhances the versatility in targeting multiple antigens but also allows the independent control of T‐cell and cancer‐cell engagement by applying pre‐targeting/post‐assembly strategy, optimizing cytotoxicity and reducing the likelihood of side effects and T‐cell exhaustion. Aiming to develop a personalized immunotherapy for MM that adapts to the unique antigen expression profiles of each patient, we validated the antitumor efficacy of MATCH across MM cell lines, MM patient‐derived samples and humanized NRG mice. Additionally, we conducted mechanism studies of MATCH, uncovering its role in inducing apoptosis in target MM cells, as well as enhancing T‐cell activation and degranulation, leading to cytotoxic granule and cytokine release. Our findings suggest that MATCH represents a promising advancement in T‐cell engaging therapy with translational potential as a next generation immunotherapy for hematologic malignancies.

## 2 Results

### 2.1 Preparation, Characterization and In Vitro Hybridization of Fab’‐MORF Conjugates

**Figure 2:**
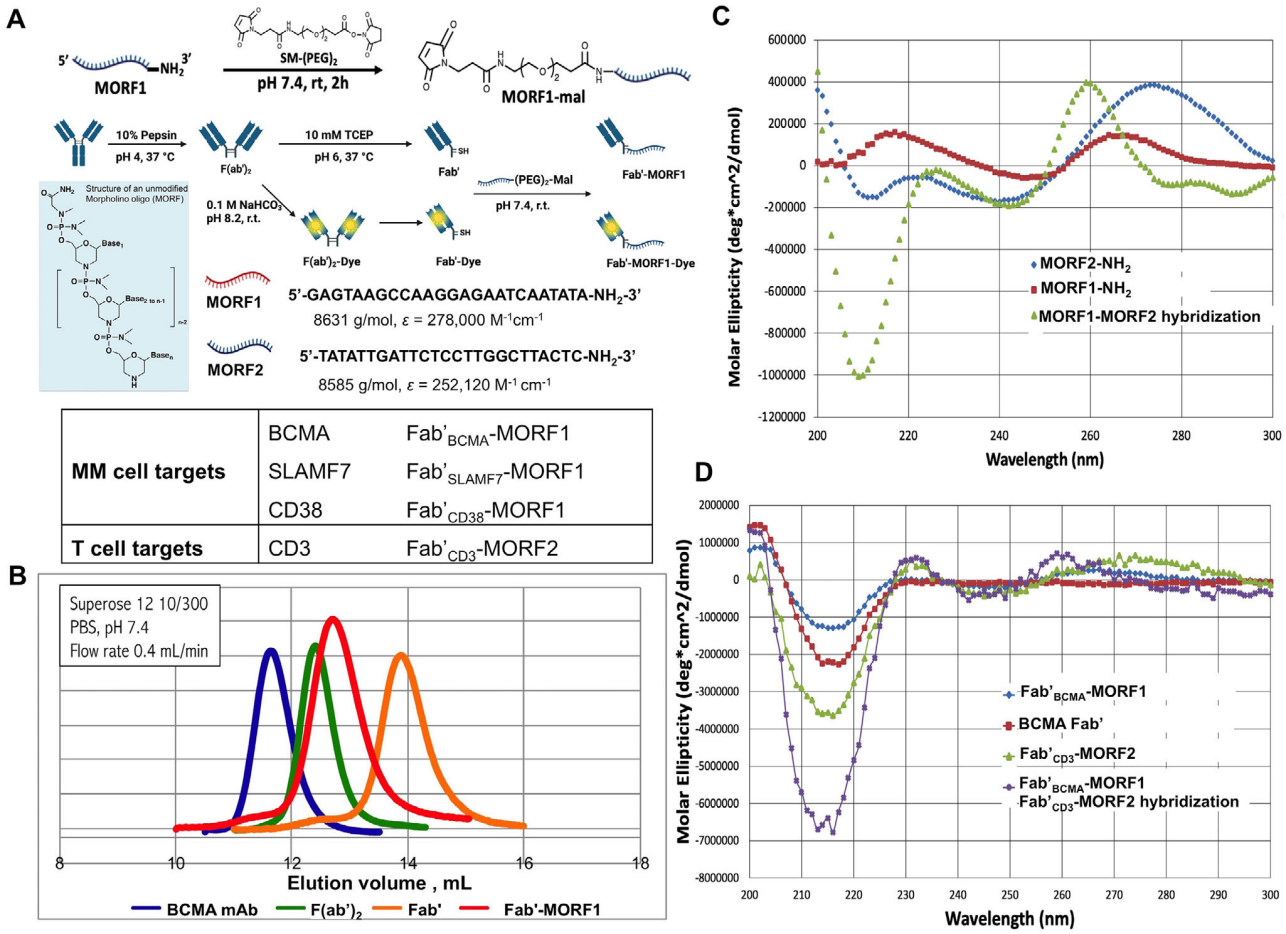
Design and synthesis of the Fab’‐MORF conjugate for MATCH platform. A) Schematic illustration of the conjugation process of maleimide terminated MORF (MORF‐mal) and Fab’‐SH, resulting in Fab’‐MORF. The sequence of the 25‐base complementary MORFs is shown. B) Example of size‐exclusion chromatography (SEC) profile of various components, including anti‐BCMA antibody, BCMA F(ab’)_2_, BCMA Fab’, and the final Fab’_BCMA_‐MORF1 conjugate, confirming successful conjugation based on volume shifts. C,D) CD spectra of the unconjugated MORFs, Fab’, the Fab’‐MORF conjugates, and the equimolar mixture of each component for analysis of hybridization. The y‐axis shows molar ellipticity (θ). When mixed, an optical signature (maxima at 260 nm, minima at 210 nm) indicating A‐form double helixes were obtained.

The MATCH platform was developed by designing and synthesizing complementary Fab’‐MORF conjugates that self‐assemble into modular T cell engagers, enabling targeted recognition of BCMA, SLAMF7, and CD38 on MM cells. As shown in **Figure** **2**A, the whole antibody was cleaved by pepsin and F(ab’)_2_ reduced by tris(2‐carboxyethyl)phosphine (TCEP). The removal of the Fc region helps minimize potential side effects.^[^ ^14^ ^]^ We conjugated MORFs to Fab’ fragments via succinimidyl‐(N‐maleimidopropionamido)‐diethyleneglycolester (SM‐(PEG)_2_) linker to create a stable, self‐assembling platform. The MORF sequences used in this study were 25‐bases in length (≈8.5 kDa) and featured a primary amine modification at the 3′ terminus for conjugation (see structure in Figure 2A). Their nucleotide composition (A/T/G/C) was optimized to ensure high binding specificity and affinity, while maintaining aqueous solubility and potentially improving pharmacokinetics by minimizing rapid renal clearance.^[^ ^12^, ^15^, ^16^ ^]^ MORF conjugates enable precise and controllable hybridization with complementary sequences, allowing for a modular and programmable design that facilitates selective target engagement. This approach offers several advantages, including high binding specificity and compatibility and synthetic flexibility with a wide range of biomolecular conjugates, supporting reliable performance in complex biological environments.^[^ ^12^, ^13^, ^17^ ^]^

Following conjugation, the formation of Fab’‐MORF nanoconjugates was confirmed by size‐exclusion chromatography (SEC), which revealed distinct elution profiles for the individual components and the final conjugates, indicating the successful generation of highly homogeneous products (Figure 2B; Figure S1, Supporting Information). The hydrodynamic diameters of the conjugates, measured via dynamic light scattering (DLS), are provided in Figure S2 (Supporting Information). The in vitro hybridization behavior of both unconjugated MORFs and Fab’‐MORF conjugates was validated using multiple analytical methods. SEC demonstrated a clear shift in elution profiles upon mixing complementary strands, consistent with hybrid complex formation (Figure S1, Supporting Information). UV–vis spectroscopy showed a hypochromic effect, evidenced by a reduction in absorbance at 260 nm, further confirming MORF1‐MORF2 pairing (Figure S3, Supporting Information). Circular dichroism (CD) spectroscopy provided additional confirmation, with spectra plotted as molar ellipticity (θ) versus wavelength showing a characteristic positive peak at 260 nm and a negative minimum near 210 nm, consistent with the formation of A‐form double helices (Figure 2C,D).

The stability of the Fab’‐MORF constructs was assessed using size‐exclusion chromatography (SEC) with a Superose S6a column. After 24 h of incubation at 37 °C in PBS, both Fab’_BCMA_‐MORF1 and Fab’_CD3_‐MORF2 retained the ability to form stable hybrids, indicating high conjugate integrity under physiological conditions (Figure S4A, Supporting Information). To further characterize the thermal behavior of the MORF1/MORF2 duplex, circular dichroism (CD) spectra were recorded for equimolar mixtures (5 µM each) in PBS (pH 7.4) across a temperature range from 25 °C to 95 °C. Upon heating, the characteristic CD profile displayed a bathochromic shift, with the positive band at 260 nm shifting toward 275 nm, suggesting conformational changes consistent with thermal denaturation (Figure S4B, Supporting Information). Molar ellipticity (θ) at 260 nm was monitored to generate a thermal melting curve, which exhibited a sigmoidal decrease with rising temperature. Fitting the data to a logistic function yielded a melting temperature (T_m_) of 63.0 °C. Comparable results were observed during the reverse temperature scan, confirming the thermal stability and reversibility of MORF1–MORF2 hybridization (Figure S4, Supporting Information).

Cell‐surface biorecognition and hybridization of Fab’‐MORF1 and Fab’‐MORF2 were further confirmed through confocal microscopy. These images provided visual evidence of in situ hybridization events at the cellular level (Figure S5, Supporting Information). Merged images revealed the contact of T cells (labeled with CellMask Green) with RPMI‐8226‐Lck‐mScarlet MM cells (red), produced yellow fluorescence at points of contact, indicative of immune synapse formation and successful hybridization between complementary MORFs. Together, these results visually validate cell‐surface assembly of the MATCH system, a critical step for effective T cell‐tumor cell engagement and functional immunological activity.

### 2.2 MATCH Effectively Activates T Cells and Induces T Cell‐Mediated Cytotoxicity in the Presence of Target Cells

**Figure 3:**
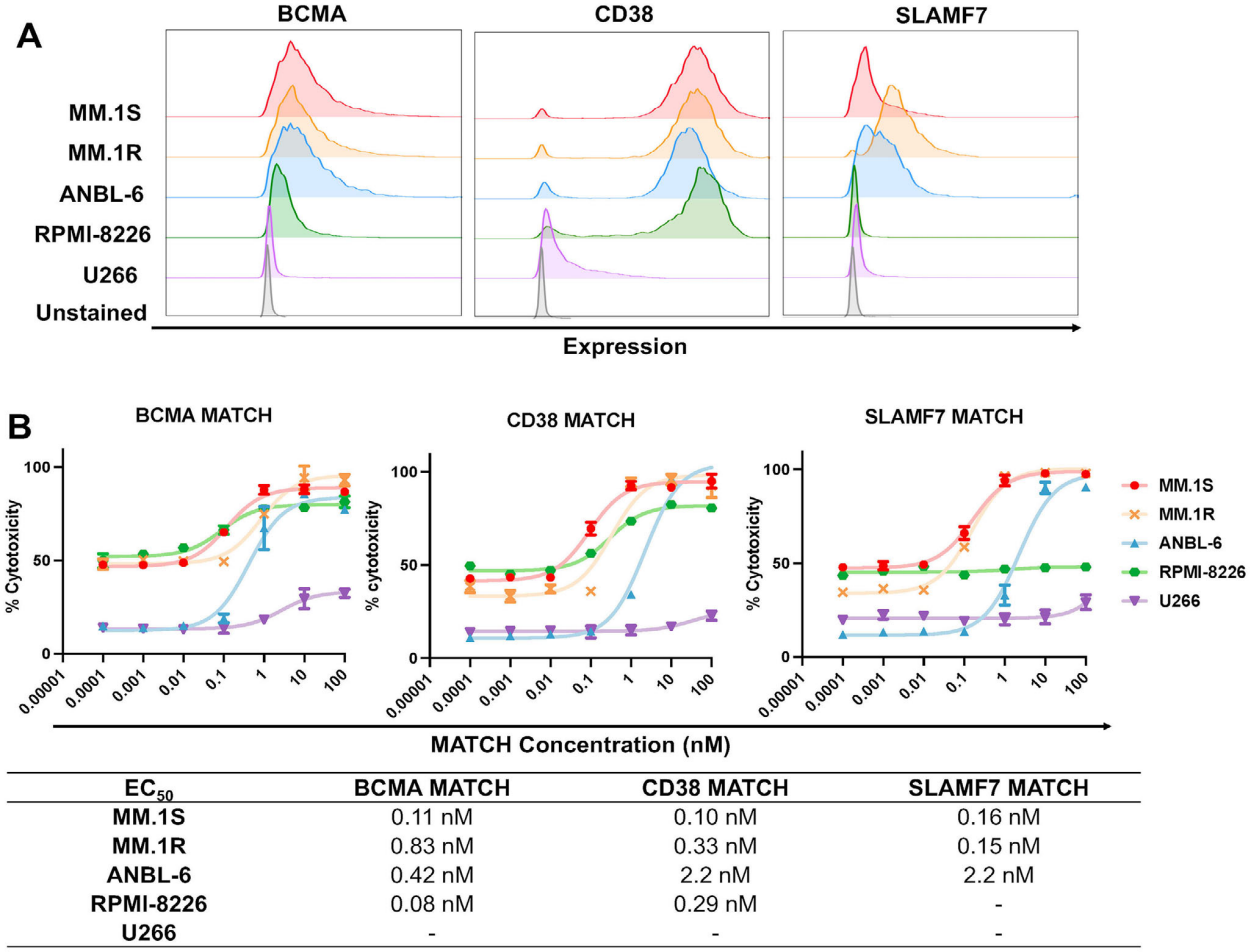
MATCH‐induced cytotoxicity against MM cells. A) Flow cytometry histograms displaying the expression levels of BCMA, CD38 and SLAMF7 on various MM cell lines. B) Cytotoxicity assay showing MATCH‐mediated killing of different MM cell lines. The EC_50_ values across different cell lines are listed in the table. Each condition was evaluated in at least three independent experiments. Drug concentrations were log‐transformed prior to analysis, and a nonlinear regression model was applied to determine EC_50_ values.

To evaluate the targeting and cytotoxic effects of MATCH, we conducted a series of in vitro assays using MM cell lines. First, flow cytometry analysis confirmed the expression level of BCMA, CD38 and SLAMF7 on five different cell lines (MM.1S, MM.1R, RPMI‐8226, ANBL‐6, and U266) (**Figure** **3**A). The results showed variable antigen expression across the cell lines, with U266 showing low expression of BCMA, CD38, and SLAMF7, and RPMI‐8226 lacked SLAMF7 expression. Flow cytometry‐based cell viability assays were used to assess the cytotoxic effect of single target BCMA/CD38/SLAMF7 MATCH on cells. As shown in Figure 3B, treatment with MATCH significantly reduced cell viability after 24 h incubation at effector‐to‐target (E:T) ratio of 1:1, with a strong dose‐dependent response in target‐specific manner. The EC_50_ values were in single digit nM or even lower in antigen‐expressing cells. Control experiments with BCMA^−^/CD38^−^/SLAMF7^−^ cells showed minimal binding and cytotoxicity, supporting the target‐selective capability of the MATCH construct. T‐cell activation and degranulation was measured by the PD‐1 and CD107a expression on the T cell surface (Figure S6, Supporting Information). Both positive population ratios increased in a dose‐dependent manner, indicating that cytotoxicity was highly related to T‐cell activation and degranulation.^[^ ^18^, ^19^ ^]^ When tumor cells lack specific antigens such as U266, T cell activation still occurred, likely due to CD3 engagement and co‐stimulation, but cytotoxicity remained minimal, suggesting limited off‐target cytotoxicity. Reduced killing may reflect the absence of a productive immunological synapse, as insufficient cell‐cell proximity or spatial organization prevents effective T cell‐mediated lysis. The cancerous B cells express co‐stimulatory receptors, such as CD80 and CD86, which may provide activation signals toward T cells (Figure S7B, Supporting Information). Notably, T‐cell activation by MATCH required the presence of target tumor cells; Fab’_CD3_‐MORF2 alone was insufficient to induce activation in T cells alone without target cells (Figure S7A, supporting information). In addition, primary T cells may have become sensitized during isolation and were cultured in the presence of CD3/CD28 activators and cytokines (IL‐2), making them more responsive to subthreshold stimulation (Figure S8, Supporting Information). These results underscore the critical importance of specific antigen targeting for effective T‐cell recruitment and potent antitumor activity.

To assess the relative contributions of each MATCH component to therapeutic activity, titration experiments were performed using Fab’_BCMA_‐MORF1 and Fab’_CD3_‐MORF2 on MM.1S cells at two different effector‐to‐target (E:T) ratios 1:3 and 3:1 representing T cell‐limited and tumor cell‐limited conditions, respectively. A more pronounced decline in cytotoxic activity was observed with decreasing concentrations of Fab’_CD3_‐MORF2, regardless of the E:T ratio (Figure S9, Supporting Information), underscoring the critical role of CD3 engagement in initiating T cell activation and subsequent tumor cell killing. In contrast, titration of Fab’_BCMA_‐MORF1 had a relatively modest impact, suggesting that once tumor cell binding is achieved, the degree of T cell engagement primarily dictates the cytolytic response. These findings highlight the potential to fine‐tune therapeutic efficacy by adjusting the dosing ratio or administration sequence of Fab’_CD3_‐MORF2 and Fab’_BCMA_‐MORF1, allowing for more precise control of T cell activation and tumor specificity.

### 2.3 T Cell Killing is Mediated by Perforin‐Granzyme Pathway

**Figure 4:**
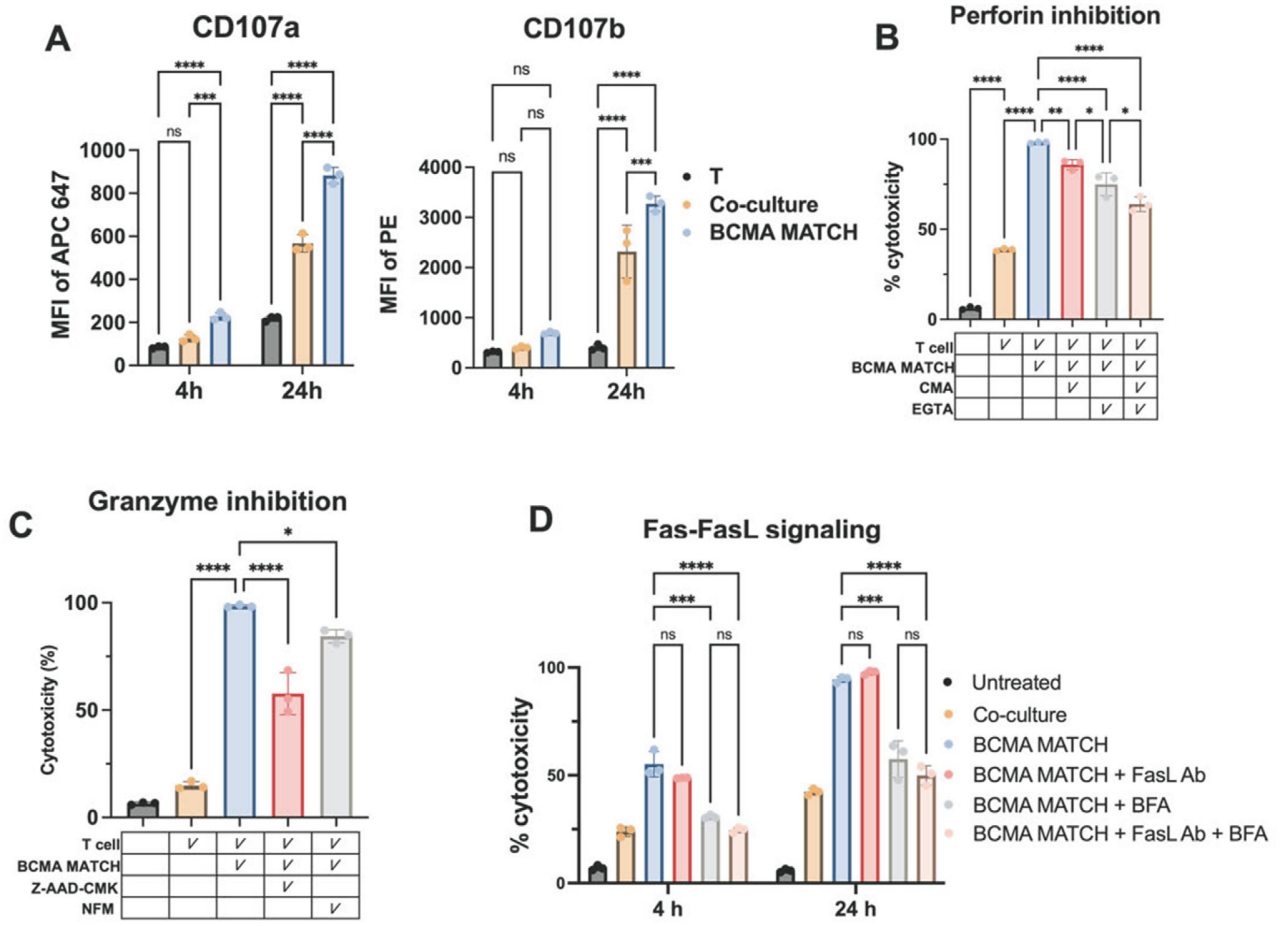
Mechanisms of MATCH‐mediated cytotoxicity. A) Expression of CD107a/b on effector T cells, indicates degranulation in response to MATCH treatment. B) Inhibition of perforin activity using concanamycin A (CMA) and calcium chelation by EGTA significantly reduces MATCH‐induced cytotoxicity. C) Targeted inhibition of granzyme B with Z‐AAD‐CMK and granzyme A with nafamostat mesylate (NFM) attenuates cell death. D) Blocking the Fas–FasL apoptotic pathway using an anti‐Fas antibody has minimal effect, while brefeldin A (BFA), a Golgi‐disrupting agent, significantly impairs cytotoxicity by interfering with granule and cytokine secretion. Statistical analysis was performed using one‐way ANOVA across treatment groups. ns = not significant; *p* < 0.05 (*), *p* < 0.01 (**), *p* < 0.001 (***), *p* < 0.0001 (****).

To determine the mechanism of T cell‐mediated cytotoxicity induced by MATCH, we first investigated expression of CD107a/b on effector T cells, markers of degranulation. After 4 and 24 h of co‐culture of T cells with MM.1S cells in the presence of BCMA‐based MATCH treatment, we observed a substantial increase in CD107a/b (**Figure** **4**A), indicating that MATCH effectively induces T cell degranulation. This prompted further investigation into the release and functional contribution of key cytotoxic granule components, including perforin, granzyme B, and granzyme A. Perforin is a pore‐forming protein that creates openings in the target cell membrane that facilitates the entry of granzymes into target cells, where they initiate apoptotic signaling cascade.^[^ ^20^ ^]^ To dissect the roles of individual cytotoxic molecules in MATCH‐mediated killing, MM.1S cells were pre‐treated with various inhibitors. Both concanamycin A (CMA), an inhibitor of vacuolar H⁺‐ATPase that blocks perforin maturation, and EGTA, a calcium chelator that disrupts calcium‐dependent granule exocytosis, significantly reduced MATCH‐induced cytotoxicity (Figure 4B).^[^ ^21^ ^]^ Additionally, specific inhibition of granzyme B using Z‐AAD‐CMK and granzyme A using nafamostat mesylate (NFM) also led to reduced target cell death (Figure 4C), demonstrating that both granzymes are critical for MATCH‐mediated cytotoxicity.^[^ ^22^ ^]^

To evaluate the contribution of the Fas–FasL (CD95–CD95L) pathway, we employed a FasL‐blocking antibody. Blocking this apoptotic axis had minimal impact on cytotoxicity (Figure 4D), suggesting that Fas‐mediated signaling is not the dominant mechanism in this context. While CD95 expression was upregulated following T cell co‐culture, and cells exhibited sensitivity to the recombinant Fas agonist CH‐11 in isolation (Figure S10, Supporting Information), blocking Fas‐FasL interactions during MATCH treatment did not alter cell death levels. This aligns with prior findings showing that B cell malignancies, such as Raji cells, may exhibit resistance to Fas‐mediated apoptosis in bispecific antibody systems.^[^ ^23^ ^]^ To further investigate the role of protein intracellular trafficking in T cell cytotoxic function, we utilized brefeldin A (BFA), which disrupts endoplasmic reticulum (ER)‐to‐Golgi transport. BFA pre‐treatment significantly reduced MATCH‐induced cytotoxicity, likely due to impaired processing and secretion of granules, cytokines and preventing membrane presentation of key effector molecules.^[^ ^22^, ^24^ ^]^ These findings indicate that while Fas–FasL interactions may play a minor supportive role, MATCH‐induced cell death relies predominantly on perforin‐granule mediated pathway.

### 2.4 MATCH Effectively Induces Apoptotic Cell Death via a Caspase‐Dependent Pathway

**Figure 5:**
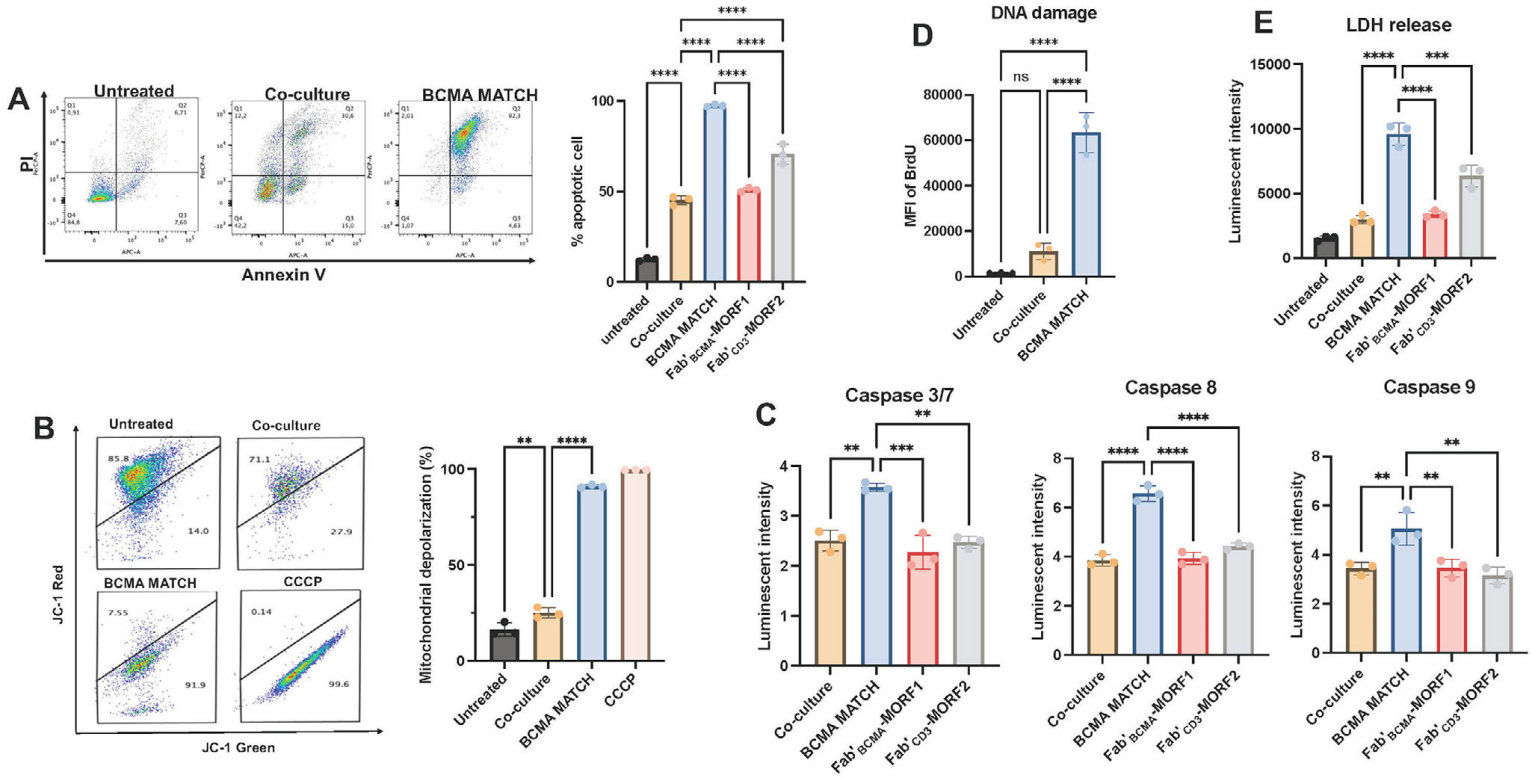
MATCH‐induced apoptosis in MM cells. A) Flow cytometry plots showing the increased percentage of apoptotic MM.1S cells following MATCH treatment compared to controls. B) Mitochondrial depolarization in target cells, indicating loss of membrane potential after treatment. C) Quantitative analysis of apoptotic markers, including caspase‐3/7, ‐8, and ‐9 activities, showing increased activation in MATCH‐treated cells. D) Assessment of DNA damage via FITC‐labeled dBrU incorporation, indicating nuclear fragmentation. E) Quantification of lactate dehydrogenase (LDH) release, reflecting membrane disruption following MATCH treatment. Statistical analysis was performed using one‐way ANOVA across treatment groups. ns = not significant; *p* < 0.05 (*), *p* < 0.01 (**), *p* < 0.001 (***), *p* < 0.0001 (****).

Granzymes are key mediators of apoptosis, operating through both caspase‐dependent and ‐independent mechanisms.^[^ ^25^ ^]^ Granzyme B is known to directly activate caspase‐3 and ‐8, the primary executioner caspases, and also cleave Bid into tBid, which translocates to the mitochondria.^[^ ^26^, ^27^ ^]^ There, tBid induces mitochondrial outer membrane permeabilization, leading to the release of cytochrome c and loss in mitochondrial membrane potential (Δψm) and formation of the apoptosome, which activates caspase‐9. This amplifies the caspase cascade, resulting in widespread activation of caspase‐3 and subsequent cleavage of cellular substrates such as inhibitor of caspase‐activated DNase (ICAD), releasing CAD to fragment nuclear DNA. This intrinsic apoptotic pathway culminates in hallmark morphological changes, such as nuclear condensation, membrane blebbing, and ultimately apoptotic cell death in MM.^[^ ^28^ ^]^ In our experiments, flow cytometric analysis using Annexin V and Propidium Iodide (PI) staining revealed a significant increase in apoptosis cells following co‐culture with T cells exposed to BCMA‐based MATCH, indicating effective induction of apoptosis via immune effector engagement (**Figure** **5**A; Figure S11, Supporting Information). As shown in Figure 5B, BCMA‐based MATCH treatment also resulted in significant mitochondrial depolarization (loss of Δψm) in target cells, indicating mitochondrial dysfunction and initiation of intrinsic apoptosis. The expression of apoptotic markers of caspase pathway, including caspase‐3/7, ‐8, ‐9, was significantly upregulated in MATCH‐treated cells (Figure 5C), confirming that the MATCH system triggers caspase‐dependent programmed cell death. The extent of DNA damage in MM cells was evaluated via TUNNEL assay using 5‐bromo‐2′‐deoxyuridine (dBrU) incorporation, a marker for DNA strand breaks. Flow cytometric analysis (Figure 5D) showed a significant increase in dBrU‐positive cells in the MATCH‐treated group compared with co‐culture, indicating extensive DNA damage in MM.1S cells following exposure to the MATCH construct. This DNA fragmentation result was consistent with apoptotic cell death. Elevated lactate dehydrogenase (LDH) release indicates membrane damage caused by perforin‐mediated pore formation upon BCMA‐based MATCH treatment (Figure 5E). Taken together, these findings indicate that MATCH‐induced cytotoxicity in MM.1S cells is primarily driven by perforin‐ and granzyme‐mediated apoptosis through a caspase‐dependent pathway.

### 2.5 T Cells can be Restimulated by MATCH to Perform Serial Killing

**Figure 6:**
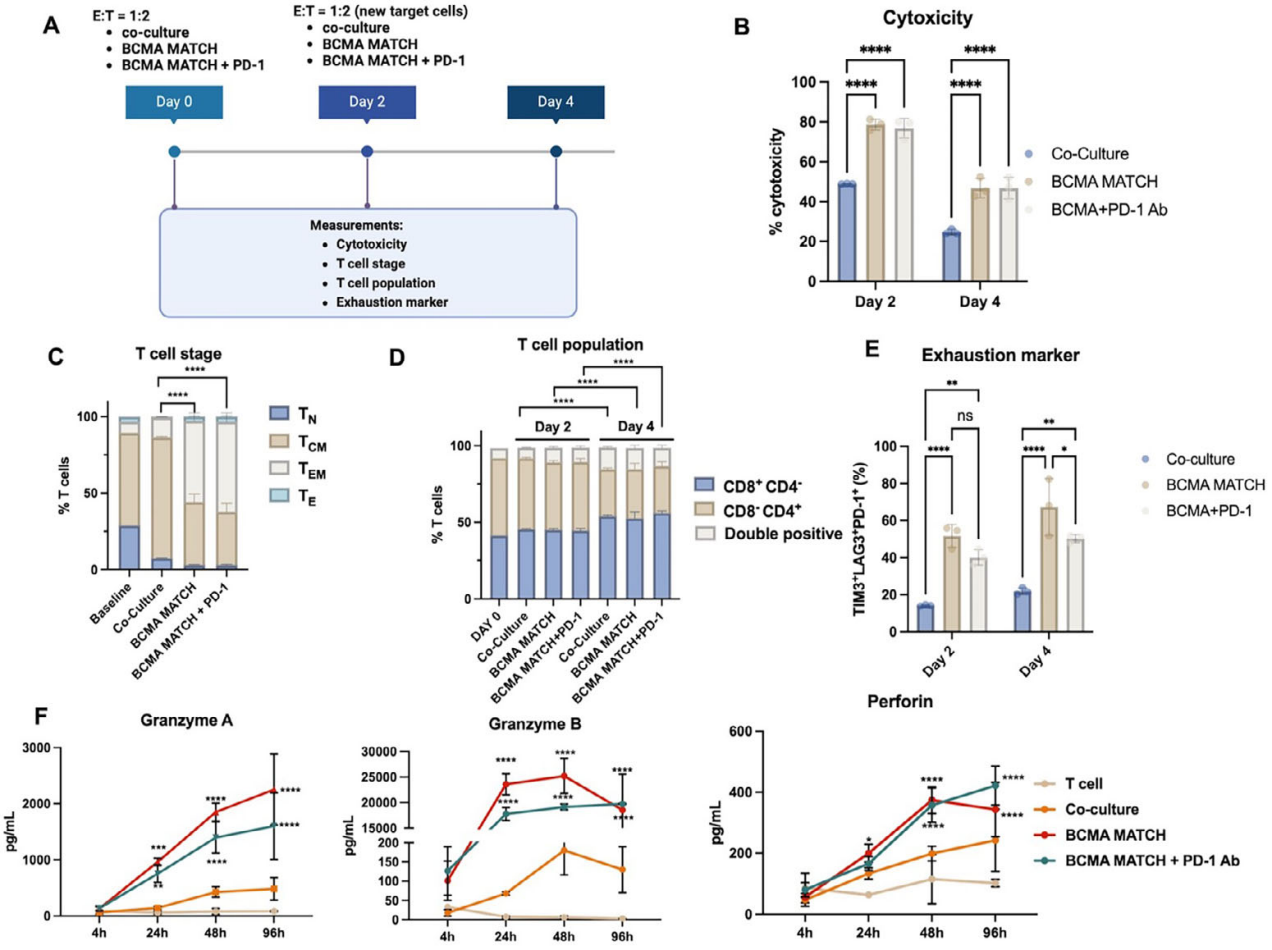
Rechallenge experiment assessing MATCH‐mediated cytotoxicity and its effects on T cells. A) Experimental timeline illustrating the schedule for rechallenge treatments and sample collection. Days 0 and 2, double the number of target cells were co‐incubated with T cells treated with BCMA‐based MATCH combined with PD‐1 blockade. Measurements on Day 2 and 4 included cytotoxicity, T cell stage (only day 4), population dynamics, and exhaustion marker expression. B) Cytotoxicity at Days 2 and 4, measured by the reduction in viable target cells. BCMA‐based MATCH and BCMA‐based MATCH + PD‐1 combination groups showed increased cytotoxicity compared to untreated controls. Each condition was tested in triplicate, and statistical analysis was performed using two‐way ANOVA followed by Tukey's post hoc test. C) Distribution of T cell differentiation stages (naïve T_N_, central memory T_CM_, effector memory T_EM_, and effector T_E_) across treatments at day 4, showing the progression of T cell maturation. MATCH‐treated groups demonstrate a shift toward memory and effector phenotypes. Each condition was tested in triplicate, and statistical analysis was performed using two‐way ANOVA followed by Tukey's post hoc test. **** indicates a statistically significant difference between T_CM_ and T_EM_ subsets, p < 0.0001. D) Analysis of T cell population. The CD8+ cell population increased on day 4 compared to day 2. **** indicates a statistically significant difference between CD4^+^CD8^−^ and CD8^+^CD4^−^ subsets, p < 0.0001. E) PD‐1^+^TIM‐3^+^LAG3^+^ triple positive cell population increased after 4 days incubation compared to untreated. With PD‐1 block, the percentage decreased in response to MATCH treatments. Each condition was tested in triplicate, and statistical analysis was performed using two‐way ANOVA followed by Tukey's post hoc test. F) Granzyme A, granzyme B and perforin concentration in cell medium was measured at different time points, indicating T cell activation. Asterisks (*) indicate statistically significant differences compared to the co‐culture group, as determined by two‐way ANOVA. ns = not significant; *p* < 0.05 (*), *p* < 0.01 (**), *p* < 0.001 (***), *p* < 0.0001 (****). [Correction added on 5 August 2025 after first online publication: Figure 6 has been updated.]

To assess whether T cells can be effectively restimulated for successive rounds of cytotoxic activity, we evaluated the impact of BCMA‐based MATCH stimulation with and without PD‐1 blockade over a 4‐day period (**Figure** **6**A). Anti‐PD‐1 antibody was used as agent to reinvigorate exhausted T cells.^[^ ^29^ ^]^ In this experiment, T cells were co‐cultured with target cells at an effector‐to‐target (E:T) ratio of 1:2 under two conditions: (i) BCMA‐based MATCH alone and (ii) BCMA‐based MATCH in combination with anti‐PD‐1 antibody. Measurements were taken at Days 2 and 4, focusing on cytotoxicity, T cell differentiation, and exhaustion marker expression. At Day 2, cytotoxicity assays demonstrated a significant enhancement in T cell killing capacity under BCMA‐based MATCH stimulation. By Day 4, flow cytometric analysis revealed a notable shift in the T cell population toward an effector memory phenotype, especially in cultures receiving the combined BCMA‐based MATCH and PD‐1 inhibition. Concurrently, these T cells exhibited a marked reduction in exhaustion marker expression (Figure 6E), indicating that PD‐1 blockade helps sustain T cell functionality over prolonged stimulation. Furthermore, quantification of cytotoxic effector molecules, granzyme A, granzyme B, and perforin, in the culture medium showed dramatic increases following MATCH stimulation (Figure 6F). However, no significant differences in the levels of these molecules were observed between the BCMA‐based MATCH alone and the BCMA‐based MATCH + anti‐PD‐1 groups. Collectively, these results demonstrate that BCMA‐based MATCH effectively enhances T cell cytotoxic function and is capable of promoting a durable effector memory phenotype, while PD‐1 blockade further prevents T cell exhaustion. This combination approach holds promise for sustaining robust and repeated immune responses in therapeutic applications.

### 2.6 MATCH Effectively Eradicates Multiple Myeloma in Ex Vivo Patient Samples

**Figure 7:**
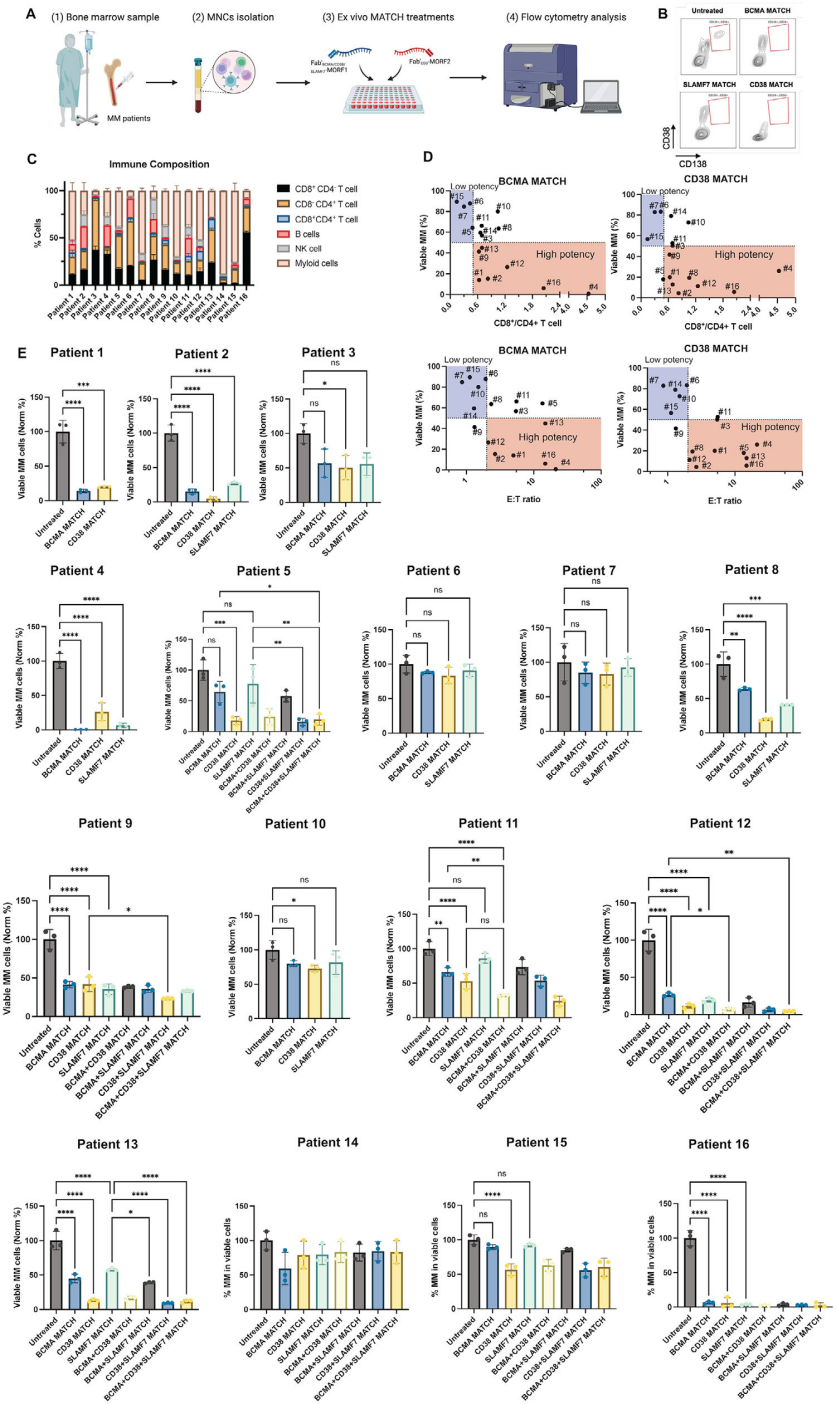
MATCH induce MM cell death in ex vivo patient samples. A) Schematic illustration and efficacy assessment of MATCH constructs from patient bone marrow samples. Peripheral blood/bone marrow is collected from patients, followed by isolation of mononuclear cells. BM‐MNCs cells and target cells are then co‐cultured in the presence of BCMA, SLAMF7, or CD38 targeted MATCH constructs, and cytotoxicity is measured via flow cytometry. B) Example of the flow results of effective eradication of MM cells in patient samples (Patient 2). C) Immune composition of patient samples (MM excluded). D) Patient sample responses to the BCMA or CD38 MATCH treatment versus the CD8+/CD4+ ratio or E:T ratio with “high potency” (red shaded region) and “low potency” (blue shaded region). E) Individual patient samples exhibited unique responses to the MATCH treatments. Each sample was tested in triplicate, and statistical analysis was performed using one‐way ANOVA. ns = not significant; *p* < 0.05 (*), *p* < 0.01 (**), *p* < 0.001 (***), *p* < 0.0001 (****).

In ex vivo studies of MM patient samples, we evaluated MATCH efficacy by measuring the frequency of MM cells (CD45^−^/CD19^−^/CD138^+^/CD38^+^) within the viable cell population for each patient.^[^ ^30^ ^]^ Patient information on cytogenetics and previous treatment regimens is shown in **Table** **1**. Briefly, patient‐derived mononuclear cells (MNCs) were isolated and then incubated with various MATCH constructs (BCMA, CD38, or SLAMF7 monospecific, BCMA‐CD38, BCMA‐SLAMF7 or CD38‐SLAMF7 bispecific or BCMA‐CD38‐SLAMF7 trispecific) in the presence autologous T cells (**Figure** **7**A). Representative flow cytometry data illustrate these assessments (Figure 7B; Figure S12, Supporting Information). Notably, after 48 h of incubation with different MATCH constructs, MNCs formed visible clusters, further highlighting their engagement and interaction (Figure S13, Supporting Information). Given the variability in patient cell populations, including immune cell composition and disease burden, the CD8^+^/CD4^+^ T cell ratio and effector‐to‐target (E:T) ratio emerged as key factors influencing MATCH efficacy (Figure 7D).^[^ ^31^, ^32^ ^]^ For instance, patients 6 and 7, who exhibited lower CD8^+^/CD4^+^ ratios and low E:T ratios, demonstrated reduced MATCH efficacy. In contrast, patients 4 and 16, who had a higher CD8^+^/CD4^+^ ratio and E:T ratio, exhibited a stronger response to MATCH treatment. Patient 10, despite having a favorable CD8^+^ T cell percentage, had a high proportion of myeloid cells, likely including suppressive tumor‐associated macrophages, which may have contributed to a diminished response (Figure S14, Supporting Information).^[^ ^33^ ^]^ Overall, MATCH treatment significantly reduced MM cell percentages compared to untreated controls in 13 out of 16 patients, underscoring its potential for effective MM targeting (Figure 7E). Individual patient responses varied depending on the MATCH construct used. For example, patient 2 demonstrated higher sensitivity to CD38‐targeted MATCH, whereas patient 4 responded more strongly to BCMA‐targeted MATCH (Figure 6E). This variability may be linked to patient‐specific genetic abnormalities and previous treatment with Daratumumab. The presence of 1q21 gain (gain of additional copies of the long arm of chromosome 1 at band q21 and t(4;14) (translocation between chromosome 4 and chromosome 14) or del(17p) (deletion of the short arm of chromosome 17) has been associated with higher BCMA expression and lower CD38 expression, whereas t(11;14) (translocation between chromosome 11 and chromosome 14) have been linked to lower CD38.^[^ ^34^, ^35^, ^36^ ^]^ Notably, the ability to tailor MATCH constructs to target specific antigens may help overcome the inherent heterogeneity of MM. This adaptability offers a promising strategy for individualized therapeutic interventions, as exemplified by patients 5, 9, 11, and 12. These findings highlight the potential for leveraging target expression profiles to optimize drug sensitivity and personalize MM treatment approaches.

**Table 1:** Information of Patients.

Patient No.	Sample Type	Gender^a)^	Age	Myeloma subtype	Cytogenetics at diagnosis^b)^	Treatment regimens^c)^
1	BM	M	64	IgA‐K	del1p, amp1q21, del16q	D‐KRd
2	BM	M	67	IgG‐K	Normal	D‐RVd
3	BM	F	70	IgG‐K	t(11;14)	D‐RVd, VenDd
4	BM	M	55	IgG‐K	t(4;14), 1q21gain, trisomy 11	VD‐PACE, D‐CyBorD, D‐RVd, mCAD, MEL
5	BM	M	74	IgG‐L	trisomy 11, t(11;14)	Smoldering myeloma – not treated
6	BM	M	60	IgG‐K	del17p, trisomy 9, 11, 17, del16q	Smoldering myeloma – not treated
7	BM	M	74	IgA‐K	Trisomy 4, 9, 11, 17	Sample obtained at diagnosis
8	BM	F	68	IgA‐K	del17p, trisomy 5, 9, 15	D‐RVd, MEL, VR maintenance, mCAD, DKd
9	BM	F	80	IgA‐K	1q21gain, trisomy 9, 14	VRd, DRd
10	BM	M	58	IgG‐L	t(11;14)	CyBorD, VRd, DPACE, MEL, Len maintenance, KPd, MEL, KPd, DRd, Abecma, VenKd, Elranatamab
11	BM	F	72	IgG‐K	del17p, del1p, 1q21gain, trisomy 11, 14, 20	Sample obtained at diagnosis
12	BM	M	62	LLC	trisomy 14	Sample obtained at diagnosis
13	BM	M	71	IgG‐L	t(11;14)	Newly diagnosed
14	BM	M	57	IgG‐L	Normal	D‐CyBorD
15	BM	M	60	IgG‐K	del13q, t(4;14)	DPACE, MEL1, MEL2, DPACE, VTd, RVd, KRd, MEL3, R, DRd, DVd, EPd, K‐mCAD
16	BM	M	72	IgG‐K	t(11;14)	Newly diagnosed

^a)^ M = male; F = female;

^b)^ amp = amplification (4 or more copies); gain = 3 copies; According to IMWG criteria for MM: High Risk: del(17p), t(4;14), t(14;16), t(14;20), amplification of 1q21, complex cytogenetics, or hypodiploidy; Intermediate Risk: t(6;14), deletion 13, or any other abnormalities not categorized as standard or high risk. Standard Risk: Normal cytogenetics, hyperdiploidy, or t(11;14);

^c)^ D = daratumumab; K = carfilzomib; R = lenalidomide; d = dexamethasone; Ven = venetoclax; VD‐PACE = bortezomib, dexamethasone, etoposide, cisplatin, cyclophosphamide, doxorubicin; Cy = cyclophosphamide; mCAD = doxorubicin, cyclophosphamide; MEL = melphalan autologous transplant.

### 2.7 MATCH Exhibits Favorable Anti‐Tumor Efficacy and Pharmacokinetics in Humanized Mouse Model

**Figure 8:**
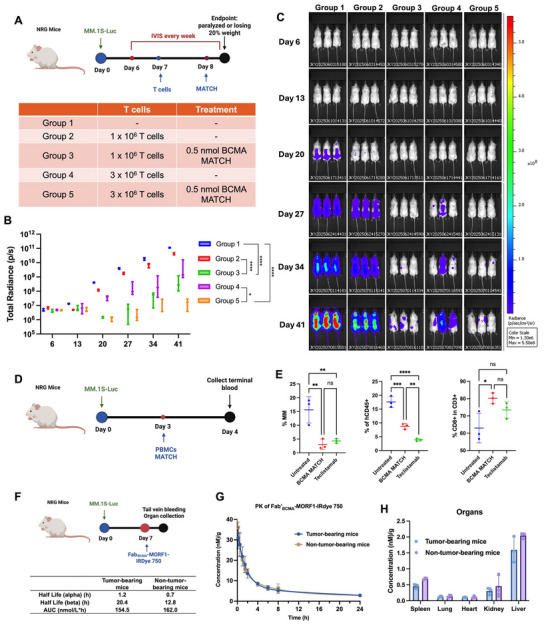
In vivo efficacy, short‐term safety, pharmacokinetics, and evaluation of BCMA‐MATCH in humanized NRG mouse models. A) Schematic of long‐term efficacy study: NRG mice were intravenously injected with MM.1S‐Luc cells (3 × 10^6^) on Day 0. T cells (1 or 3 × 10^6^) were administered on Day 7, followed by BCMA‐MATCH treatment (0.5 nmol equivalent) on Day 8. Mice were monitored weekly by IVIS imaging. B) Quantification of total body radiance over time demonstrated significant tumor suppression in the MATCH‐treated groups compared to both T cell‐only and untreated controls (n = 3). Statistical significance was assessed using two‐way ANOVA followed by Tukey's post hoc test. C) IVIS images over time show marked tumor burden reduction in MATCH‐treated groups relative to controls. D) Schematic of short‐term toxicity study: MM.1S‐Luc–engrafted mice were treated with PBMCs and BCMA‐MATCH on Day 3 and analyzed on Day 4. E) Flow cytometry analysis at 24 h post‐treatment revealed a significant reduction in tumor burden in both the BCMA‐MATCH and Teclistamab groups compared to the control (n = 3). Statistical significance was assessed using one‐way ANOVA. A modest decrease in the percentage of circulating human CD45⁺ PBMCs was observed, accompanied by an increased proportion of CD8⁺ T cells within the T cell compartment in MATCH‐treated mice. F) Schematic of pharmacokinetics study. G) Pharmacokinetics of Fab’_BCMA_‐MORF1 in MM.1S tumor‐bearing NRG mice reveals biphasic clearance with t_1/2_α = 1.2 h and t_1/2_β = 20.4 h; clearance was faster in non–tumor‐bearing mice (t_1/2_β = 12.8 h). H) Biodistribution analysis at 24 h post‐injection shows minimal accumulation of Fab’_BCMA_‐MORF1–IRDye 750 in liver and kidneys, with no abnormal retention. ns = not significant; *p* < 0.05 (*), *p* < 0.01 (**), *p* < 0.001 (***), *p* < 0.0001 (****).

To evaluate the in vivo efficacy of MATCH, luciferase‐expressing multiple myeloma cells (MM.1S‐Luc, 3 × 10^6^) were intravenously injected into NRG mice on Day 0. Mice were randomized into five treatment groups (n = 3 per group): Group 1 received no treatment (control); Group 2 received 1 × 10^6^ T cells; Group 3 received 1 × 10^6^ T cells plus MATCH (0.5 nmol equivalent of MORF1/MORF2); Group 4 received 3 × 10^6^ T cells; and Group 5 received 3 × 10^6^ T cells plus MATCH (0.5 nmol equivalent of MORF1/MORF2). T cells were administered on Day 7, followed by BCMA‐based MATCH treatment on Day 8. Mice were monitored longitudinally by in vivo bioluminescence imaging (BLI) using the IVIS Spectrum system (PerkinElmer). Imaging was conducted once per week beginning from Day 6 (**Figure** **8**A). Mice were anesthetized and injected intraperitoneally with D‐luciferin (3 mg/mouse), and total flux (photons/sec) from the whole body was quantified using Living Image software. Tumor progression was monitored over time, and animals were sacrificed upon reaching endpoint criteria, defined as hind‐limb paralysis or a 20% loss in body weight. Quantitative analysis of total radiance demonstrated that mice treated with BCMA‐based MATCH exhibited significantly reduced tumor burden compared to control groups, including both untreated mice and those receiving the same number of T cells without MATCH treatment (Figure 8B). IVIS images over the course of treatment from each group are shown, with lower radiance signals correlating with suppressed tumor growth (Figure 8C). No significant body weight loss was observed in the MATCH‐treated groups throughout the study, indicating good tolerability and the absence of observable systemic toxicity. In contrast, mice in the tumor progression control group exhibited marked weight loss and were euthanized upon reaching the 20% body weight loss endpoint, in accordance with ethical guidelines. These data indicate that MATCH treatment effectively limits tumor progression in vivo.

Short‐term toxicity and immunological impact were evaluated in a humanized NRG mouse model. A single injection of human PBMCs followed by 1 nmol BCMA‐MATCH resulted in a significant reduction in MM.1S tumor burden compared to untreated controls and exhibited efficacy comparable to teclistamab within 24 h (Figure 8D,E). BCMA‐MATCH treatment led to a modest decrease in circulating human PBMCs, though levels remained higher than those observed in the teclistamab‐treated group. Additionally, a greater proportion of CD8⁺ T cells was detected within the T cell population following BCMA‐MATCH administration. Notably, no signs of acute cytokine release or observable systemic toxicity were detected within the 24 h window, indicating that transient T cell activation by MATCH is both effective and well tolerated in vivo.

Pharmacokinetic analysis of Fab'BCMA‐MORF1 in tumor‐bearing NRG mice demonstrated a biphasic clearance profile, characterized by a rapid distribution phase (t_1/2_α = 1.2 h) followed by a slower elimination phase (t_1/2_ β = 20.4 h) (Figure 8F,G). In comparison, non–tumor‐bearing mice exhibited slightly faster clearance, with an elimination half‐life of 12.8 h. Biodistribution studies at 24 h post‐injection revealed minor accumulation in the liver and kidneys, with no evidence of abnormal retention, indicating a favorable clearance profile supportive of future clinical translation (Figure 8H; Figure S15, Supporting Information).

## 3 Discussion

MM remains a challenging malignancy due to its inherent heterogeneity, capacity for immune evasion, and the development of drug resistance.^[^ ^37^ ^]^ While single‐antigen therapies have demonstrated initial clinical efficacy, they are often undermined by antigen downregulation, mutation, or loss, ultimately resulting in disease relapse. To overcome these limitations, researchers have increasingly turned to multi‐targeting strategies that address more than one antigen simultaneously.^[^ ^38^ ^]^ Such approaches hold promise for improving the durability of antitumor responses, reducing relapse rates, and better managing resistant disease. In this study, we introduce the MATCH (Multi‐Antigen T Cell Hybridizer) platform as a novel and modular approach to bispecific T cell engager therapy. MATCH enables flexible assembly of antigen‐targeting Fab′ fragments with T cell–engaging components via oligonucleotide‐guided hybridization. This system supports simultaneous or switchable targeting of multiple antigens, such as BCMA, CD38, and SLAMF7, on MM cells. Compared to traditional single‐antigen approaches, MATCH offers two key advantages: 1) the capacity to reduce immune escape by co‐targeting redundant or complementary antigens, and 2) the potential for customization to match a patient's unique tumor profile—aligning with the principles of precision medicine.

In line with these findings, our study demonstrates that the MATCH platform's modular design can facilitate the formation of immune synapses between T cells and MM cells, effectively activating T cells and promoting cytotoxic degranulation. One of the key features of MATCH is the ability to exert the killing function only when target expression is present. By activating perforin‐granzymes pathway in T cells, and caspase‐dependent apoptosis pathway in MM.1S cells, MATCH has the potential to maximize T cell cytotoxicity and sustain an effective immune response against MM cells. Ex vivo experiments using MM patient‐derived samples revealed that tumor antigen heterogeneity and individual T cell subsets can influence the therapeutic efficacy of MATCH, highlighting a crucial need for personalized treatment strategies. Our findings further highlight how patient‐specific factors, particularly immune cell composition and disease burden, can profoundly influence therapeutic outcomes. Notably, the CD8^+^/CD4^+^ T cell ratio, as well as the effector‐to‐target (E:T) ratio, emerged as key determinants of MATCH efficacy. Overall, MATCH treatment yielded significantly reduced MM cell percentages compared to untreated controls in 13 out of 16 patients. Individual patient responses varied depending on the specific MATCH construct used, which might relate to previous treatment and cytogenetic abnormalities. To support clinical translation, we incorporated a long‐term efficacy study in MM.1S‐Luc–bearing NRG mice. Weekly IVIS imaging demonstrated that BCMA‐MATCH led to sustained tumor suppression over time. Mice receiving 3 × 10^6^ T cells plus MATCH exhibited the greatest reduction in tumor burden without significant weight loss or toxicity, confirming the platform's durable therapeutic effect and good tolerability in vivo. In vivo pharmacokinetic profiling of Fab’_BCMA_‐MORF1 revealed a biphasic clearance pattern with a rapid distribution phase and a slower elimination phase. Biodistribution studies indicated no abnormal retention in organs, suggesting a favorable clearance profile for clinical translation. No signs of cytokine release syndrome (CRS) or overt systemic toxicity were observed, indicating that transient T cell activation by MATCH is both potent and well tolerated.

Despite these encouraging findings, several limitations must be addressed in future studies. First, in vitro and ex vivo assays do not fully capture the complexity of the bone marrow microenvironment or the immunosuppressive factors present in MM. We acknowledge the need for comprehensive evaluation of the in vivo metabolic behavior, immunogenicity, and long‐term biosafety of the MATCH system. Although our short‐term toxicity and biodistribution data are encouraging, extended studies are essential to fully characterize the pharmacodynamics, clearance pathways, and potential accumulation of MATCH components in non‐target tissues. Second, antigen expression on MM cells is highly dynamic and can be influenced by various factors, including disease progression, treatment history, and the tumor microenvironment. Receptor internalization following antibody or Fab’ fragment binding can significantly affect therapeutic efficacy. Upon ligand engagement, certain surface antigens undergo rapid endocytosis, which may reduce the availability of binding sites for subsequent MATCH component engagement or limit the formation of stable immune synapses. This internalization could impair sustained T‐cell activation and reduce cytotoxic potential, particularly in sequential or low‐dose treatment regimens. Strategies such as selecting antibodies that promote slower internalization, engineering receptor‐stabilizing mutations, or fine‐tuning the timing between pre‐targeting and T cell engager delivery may help preserve surface expression and improve therapeutic durability. Lastly, as a modular and customizable therapeutic, MATCH introduces new challenges in terms of large‐scale conjugation, oligonucleotide synthesis, and batch‐to‐batch consistency of Fab’‐MORF conjugates. Future work will focus on optimizing the scale‐up and manufacturing processes, while advancing stability and storage formulations compatible with clinical use. From a translational perspective, MATCH presents a unique opportunity to construct tri‐ or even tetra‐specific engager systems, beyond the capabilities of conventional antibody engineering, by leveraging its programmable oligonucleotide assembly. This could be especially powerful for treating highly heterogeneous malignancies, where tumor subclones often express divergent antigen profiles. Moreover, the MATCH framework could be expanded for immune modulation beyond cytotoxic T‐cell redirection, including combinatorial engagement of immune checkpoints or cytokine neutralization. High‐throughput screening of MATCH constructs in PBMC–tumor co‐culture systems may also enable rapid identification of synergistic target combinations and predictive biomarkers of response. Together, these directions support MATCH as not only a therapeutic tool but a flexible discovery platform for precision immunotherapy.

In summary, the MATCH platform represents a promising advancement in T‐cell engager therapy. By combining the flexibility of modular assembly with multi‐antigen targeting, MATCH addresses several limitations of current T‐cell therapies. Our findings underscore the potential of MATCH as a next‐generation immunotherapy capable of tailoring immune responses to the unique antigen profiles of individual patients.

## 4 Conclusion

In this study, we developed and evaluated the MATCH platform as a next‐generation, modular T cell engager system designed to address the limitations of current single‐antigen immunotherapies. By leveraging oligonucleotide‐guided self‐assembly, MATCH enables flexible, multi‐antigen targeting with high specificity, tunable engagement, and enhanced potential for personalized therapy. Our findings demonstrate that MATCH effectively bridges T cells and MM cells, promotes immune synapse formation, activates cytotoxic granule pathways, and induces caspase‐dependent apoptosis. Both, ex vivo patient‐derived sample analysis and in vivo studies in humanized mouse models confirm the therapeutic potential of MATCH, with evidence of significant tumor suppression, favorable pharmacokinetics, minimal toxicity, and immune selectivity. Importantly, the modularity of the MATCH platform supports the rapid development of customized engagers tailored to individual patient tumor profiles—aligning with the principles of precision oncology. This platform also lays the groundwork for future expansion into tri‐ or tetra‐specific constructs and high‐throughput target screening applications. Although further investigation is warranted to evaluate long‐term safety, antigen internalization, and potential off‐tumor effects, MATCH represents a promising step toward safe, adaptable, and potent T cell–based immunotherapy. With continued optimization and validation, MATCH holds strong translational potential for improving outcomes in patients with relapsed, refractory, or antigen‐heterogeneous multiple myeloma.

## 5 Experimental Section

### Design and Synthesis of Fab’‐MORF Conjugates

Monoclonal anti‐BCMA antibody (Biointron), anti‐CD38 antibody (Daratumumab, Darzalex, Janssen Biotech), anti‐SLAMF7 antibody (Bristol Myers Squibb), anti‐CD3 antibody (Ichorbio) were first digested into F(ab’)_2_ with 10% (w/w) pepsin (Sigma–Aldrich) in 0.1 M citric buffer (pH 4.0) at 37 °C for at least 1 h. Then, F(ab’)_2_ was reduced to Fab’‐SH using 10 mM TCEP (Sigma–Aldrich) in 0.1 M citrate‐phosphate buffer (pH 6.0) at 37 °C. In parallel, the end group of MORF1 (3′‐amine‐derivatized 25‐mer phosphorodiamidate morpholino oligonucleotide, Gene Tools) was converted to maleimide group by reaction with 50x excess of SM(PEG)_2_ (Thermo Fisher Scientific). Excess SM(PEG)_2_ was removed by ultracentrafiltration (Ultracel 3,000 Da MWCO, Millipore) with phosphate‐buffered saline (PBS, pH 7.4) washing. Freshly reduced Fab’‐SH was used to conjugate with 3′‐maleimide‐derived MORF1 with the molar ratio of 1: 1.1. Each step was monitored using SEC equipped with Sephacryl S200 column, using filtered 1× PBS at pH 7.4 as the eluent to ensure the reaction was completed. Fab’ equivalent concentration and MORF1 content in Fab’‐MORF1 were determined using bicinchoninic acid (BCA) protein assay (Pierce) and UV–vis spectrophotometry (Nanodrop, ND‐1000 Spectrophotometer, solution in 0.1 N HCl at 260 nM, molar absorption coefficient 278 000 M^−1^cm^−1^ for MORF1, 252 210 M^−1^cm^−1^ for MORF2).

To prepare Fab’‐MORF1‐IRdye 750, Fab’‐MORF2‐Cy3, or Fab’‐MORF1‐Cy5, F(ab’)_2_ was labeled with IRdye 750 monosuccinimidyl ester (IRdye 750 NHS Ester, LI‐COR), cyanine 3 monosuccinimidyl ester (Cy3‐NHS Ester, Lumiprobe) or cyanine 5 monosuccinimidyl ester (Cy5‐NHS Ester, Lumiprobe) by reaction with lysine side chains at room temperature. F(ab’)_2_‐IRdye 750, F(ab’)_2_‐Cy3 or F(ab’)_2_‐Cy5 were purified using a PD 10 column (Cytiva) to remove the unreacted dye, then worked up as described above.

### Detection of Hybridization by UV–Visible Spectroscopy

Hybridization between Fab’‐MORF1 and Fab’‐MORF2 was assessed by analyzing the hypochromic effect using a UV–vis spectrophotometry (Nanodrop, ND‐1000 Spectrophotometer). Fab’‐MORF1 and Fab’‐MORF2 were each dissolved in PBS (pH 7.4) at a concentration of 2.5 µM (MORF equivalent) and mixed at varying molar ratios while keeping the total MORF concentration constant at 2.5 µM. For example, an 80:20 Fab’‐MORF1 to Fab’‐MORF2 mixture was prepared by combining 0.8 mL of Fab’‐MORF1 (2.5 µM) with 0.2 mL of Fab’‐MORF2 (2.5 µM). The absorbance at 260 nm was measured to detect hybridization‐induced hypochromicity, indicative of base stacking and duplex formation. All measurements were performed in triplicate to ensure reproducibility.

### Dynamic Light Scattering (DLS)

The hydrodynamic diameters of Fab’‐MORF1 and Fab’‐MORF2, along with their respective precursors (whole antibody and F(ab’)_2_), were characterized by dynamic light scattering (DLS, Malvern). Measurements were performed at room temperature in PBS (pH 7.4). Samples were filtered through a 0.22 µm syringe filter prior to analysis.

### Circular Dichroism (CD) Spectrometry

CD spectra were acquired using an Aviv 62DS CD spectrometer equipped with thermoelectric temperature control (Aviv Biomedical, Lakewood, NJ). Standard spectral scans (excluding thermal melting) were conducted at 25 °C, recording from 300 to 200 nm at 1 nm intervals (bandwidth = 1 nm, 2 sec per step). Samples were prepared in PBS (pH 7.4) at 50 µM MORF‐equivalent concentrations. All samples were filtered through a 0.22 µm membrane and loaded into a 0.1‐cm path length quartz cuvette. Background spectra (PBS only) were subtracted from each sample spectrum, and data from three consecutive scans were averaged. For thermal melting analysis, MORF1 and MORF2 were mixed in an equimolar ratio (5 µM each, based on MORF equivalents) and incubated for 1 h at room temperature in PBS (pH 7.4). After filtration, the mixture was transferred to a 1‐cm path length quartz cuvette. A forward temperature scan was first performed from 25 to 95 °C in 2 °C increments, allowing 2 min equilibration followed by 30 sec data acquisition at each step. A reverse scan followed, cooling from 95 to 25 °C in 10 °C decrements, with 5 min equilibration and 30 sec data acquisition per step.

CD signal at 260 nm was monitored (n = 3), and observed ellipticity (θ_obs_) was converted to molar ellipticity (θ) using the formula: θ = θ_obs_ / (l × c), where *l* is the optical path length in cm, and *c* is the molar concentration (MORF equivalent). To determine the melting temperature (T_m_) of MORF1‐MORF2 hybridization, θ (at 260 nm) was plotted as a function of temperature (T) and fitted to a four‐parameter logistic model using GraphPad Prism.

### Fab’‐MORF1 and Fab’‐MORF2 In Vitro Hybridization

MM.1S cells were first treated with 50 nM Fab’_BCMA_‐MORF1‐Cy5 for 30 min at 4 °C, and T cells were treated with 50 nM Fab’_CD3_‐MORF2‐Cy3 for 30 min at 4 °C. Then, Fab’‐MORF solution was removed, and cells were washed with cold PBS. MM.1S and T cells were further co‐cultured together for 30 min at 37 °C in 8 well confocal chambers prior to confocal visualization.

### Multiple Myeloma Cell Line Antigen Profiling

The human cell lines MM.1S (RRID:CVCL_8792), MM.1R (RRID:CVCL_8794), RPMI‐8226 (RRID:CVCL_0014) and U266 (RRID:CVCL_0566) were purchased from American Type Culture Collection (ATCC), ANBL‐6 (RRID:CVCL_5425) was obtained from Dr. Diane Jelinek (Mayo Clinic). All cell lines have been authenticated and confirmed free of contamination. The cell lines were cultured at recommended conditions in advised media from ATCC. MM.1S, MM.1R and RPMI‐8226 cell line were cultured in RPMI 1640 medium (Gibco) supplemented with 10% fetal bovine serum (FBS), penicillin (200 U mL^−1^), and streptomycin (200 µg mL^−1^) at 37 °C with 5% CO_2_. ANBL‐6 was maintained in RPMI 1640 complete medium with 2 ng mL^−1^ IL‐6. Surface expression of BCMA, CD38 and SLAMF7 antigens on these cells were evaluated by flow cytometry (BD FACSCanto II). Briefly, 5 × 10^4^ cells were incubated with fluorescein‐labeled primary antibodies in PBS with 2% FBS at 4 °C for 30 min. Antigen expression was quantitated by geometric mean averages of labelled cells. It use all these cells to capture the genetic, phenotypic, and therapeutic heterogeneity of multiple myeloma and ensure MATCH efficacy across clinically relevant subtypes.

### Flow Cytometry‐Based T Cell Activation and Cytotoxicity Assays

CFSE‐labeled target cells were cocultured with healthy donor T cells in a 96‐well plate in a E:T ratio of 1:1 for 24 h at 37 °C dosed with MATCH from 0.001 to 100 nM. To investigate a possible sensitization of cancer cells to CD95‐induced apoptosis, cells were incubated with anti‐CD95 monoclonal IgM antibody CH‐11 (1 µg mL^−1^; MBL Life Science) for 24 h. Then, cells were collected, washed with PBS, and the ratio of cells positive for CFSE and viability dye was analyzed for cytotoxicity by flow cytometry. Negative CFSE population (T cells) was analyzed by PD‐1 positive and CD107a/b positive percentage. Each analysis was performed in triplicate.

### Apoptosis Assay

Annexin V‐APC (Biolegend) and Propidium iodide (PI, Invitrogen) staining were performed. Cocultured target cells and T cells were incubated with Fab’‐MORF1 and Fab’‐MORF2 at 10 nM of MORF. After 24 h incubation, cells were collected, stained with annexin V‐APC and PI for 20 min at 4 °C. The percentage of target cells positive for both annexin V and PI was quantified using flow cytometry.

### Antigenic Re‐Stimulation Assay and Immunophenotyping of T Cells

Cells were plated in a 96 well plate (E:T = 1:2). On day 0, MM cells and T cells were co‐cultured with different treatments: BCMA‐based MATCH and BCMA‐based MATCH with anti‐PD‐1 antibody. On day 2, cells were collected and analyzed using different markers with flow cytometry. Remaining T cells were recultured with newly added MM.1S cells on day 2 and analyzed on day 4 (stimulation cycle 2). On days 2 and 4, cultures were stained with DAPI Viability Dye for viability and fluorescence‐labeled antibodies against T cell population markers: CD5‐PE, CD4‐BUV737, CD8‐PerCP; T cell exhaustion markers: PD‐1‐BV786 (BD Bioscience), Tim‐3 (CD366)‐BB515 (BD Bioscience), and LAG‐3‐PE Dazzle 594 (BioLegend); and T cell stage markers: CD62L‐BV650 (BioLegend) and CD45RO‐APC (BioLegend). The cell medium was analyzed for granzymes and perforin using Legendplex assay (BioLegend).

### Mitochondrial Depolarization

To assess mitochondrial depolarization, cells were stained with JC‐1 dye (Thermo Fisher Scientific), a fluorescent probe commonly used to detect changes in mitochondrial membrane potential. Under normal conditions, JC‐1 accumulates in the mitochondria and forms aggregates, emitting red fluorescence. When mitochondrial depolarization occurs, JC‐1 remains in its monomeric form in the cytoplasm, emitting green fluorescence. In this assay, cells were treated with carbonyl cyanide m‐chlorophenyl hydrazone (CCCP, Thermo Fisher Scientific), a mitochondrial uncoupler, as a positive control to induce mitochondrial depolarization. Following treatment, cells were incubated with JC‐1 dye, and the fluorescence shift from red to green was measured by flow cytometry or fluorescence microscopy. The ratio of red to green fluorescence was used to quantify mitochondrial depolarization, with a lower red/green ratio indicating increased depolarization.

### Fas‐FasL Binding, Granzyme A, Granzyme B and Perforin Inhibition Assays

To determine the role of various cytotoxic pathways in MATCH‐mediated killing of MM cells, specific inhibitors were used prior to MATCH treatment. For FasL inhibition, MM cells were pre‐treated with anti‐FasL antibody (BioLegend, Clone: NOK‐1) and brefeldin A (BFA, BioLegend). Granzyme activity was blocked by treating cells with nafamostat mesylate (NFM, MedchemExpress) for granzyme A inhibition and Z‐AAD‐CMK (MedchemExpress) for granzyme B inhibition. To assess perforin's role, concanamycin A (CMA) and EGTA were used to inhibit perforin function. After pre‐treatment with each inhibitor, MATCH was added, and cell viability was assessed to evaluate the reduction in cytotoxicity.

### Intracellular Caspase Activity

To measure caspase‐3/7, ‐8, and ‐9 activity, it utilized the luminescent caspase assay kits from Promega, specifically designed for the quantitative detection of caspase activation in cell lysates. The Caspase‐Glo 3/7, Caspase‐Glo 8, and Caspase‐Glo 9 kits were used according to the manufacturer's instructions. Briefly, cells were seeded and treated under experimental conditions, then washed and incubated with the respective Caspase‐Glo reagent for 1 h. Luminescence was measured using a microplate reader, with higher luminescent values indicating greater caspase activity.

### Human PBMCs and Patient Samples

Human PBMCs (peripheral blood mononuclear cells) were purchased from ARUP (Salt Lake City, UT). Healthy donor (HD) T cells were isolated from PBMCs using the Human T Cell Isolation Kit (STEM‐CELL Technologies) and cultured in StemCell T cell media supplemented with CD3/CD28 mAb cocktail and recombinant IL‐2 based on manufacturer recommendations. MM patient bone marrow samples were obtained from the Biobank of the Division of Hematology and Hematologic Malignancies at the Huntsman Cancer Institute (Approval number: IRB 00045880). Every patient gave consent through a research protocol by University of Utah Institutional Review Board. For primary multiple myeloma samples, bone marrow (BM) aspirates were collected, and BM mononuclear cells (BMMNC) were isolated using Ficoll density gradient centrifugation with SepMate (StemCell). Cells were counted and seeded into U‐bottom 96‐well plate. The number of cells collected varied from patient‐to‐patient, ranged from 5 × 10^5^ to 1 × 10^7^ cells per well. Unfractionated BMMNC cells allowed the use of the patient's own endogenous T cells to directly measure the efficacy of MATCH against patient's cancerous B cells. Primary cells were seeded in triplicates in 96‐well plate and treated with MATCH, followed by 48 h incubation. Multiparametric flow cytometric immunophenotyping was performed in these primary samples using monoclonal antibodies against either MM markers: CD38‐PE, CD138‐FITC, CD45‐PerCP, BCMA‐APC, and CD19‐BV785, and non‐MM markers: CD3‐BV605, CD4‐BV650, CD8‐BV510, CD56‐PE/Cy7, CD19‐BV785, CD45‐PerCP, CD16‐APC/Fire750, CD11b‐BUV395, and CD235a‐Alexa Flour 700 (BioLegend). The relative cell viability was measured using DAPI.^[^ ^30^, ^32^ ^]^

### In Vivo Imaging System (IVIS) Imaging

All experiments involving animals were performed according to the protocol approved by the Institutional Animal Care and Use Committee (IACUC) of the University of Utah (Approval number: 00002405). To evaluate tumor progression and therapeutic efficacy, bioluminescence imaging was performed using an in vivo imaging system (IVIS Spectrum, PerkinElmer). NRG (NOD‐Rag1^−/−^IL2Rγ^null^) mice were intravenously injected with 3 × 10^6^ MM.1S‐Luc cells. Tumor engraftment was monitored using IVIS 6 days post‐injection. Mice were randomized into five groups (n = 3 per group): Group 1 received no treatment; Group 2 and Group 4 received 1 × 10^6^ and 3 × 10^6^ human T cells, respectively; Group 3 and Group 5 received T cells (1 × 10^6^ and 3 × 10^6^) combined with 0.5 nanomolar equivalents of BCMA‐based MATCH (pre‐mixed before injection). T cells were administered on Day 7, and treatments were administered intravenously on Day 8 post‐tumor inoculation. For bioluminescent imaging, mice were anesthetized using isoflurane and intraperitoneally injected with D‐luciferin, sodium salt (150 mg k^−1^g, GoldBio) 15 min prior to image acquisition. Whole‐body luminescence was captured using Living Image software (PerkinElmer) under auto‐exposure settings. Quantitative analysis of total radiance (photons/sec/cm^2^/sr) was performed by drawing identical regions of interest (ROIs) over each mouse and normalizing signal intensity over time. Tumor burden was monitored every week post‐treatment. All image acquisition and analysis settings were kept consistent across groups.

### PK study of Fab’_BCMA_‐MORF1

The pharmacokinetics of Fab’_BCMA_‐MORF1 was evaluated in NRG mice using an IRDye 750‐labeled version of the molecule for fluorescent tracking. Tumor‐bearing and non‐tumor bearing NRG mice (n = 3) were administered a single intravenous dose of IRDye 750‐labeled Fab’_BCMA_‐MORF. Blood samples were collected at predetermined time points post‐administration (0.17, 0.5, 1, 1.5, 2, 4, 6, 8, and 24 h) via tail vein bleeding. Plasma was separated by centrifugation and stored at 4 °C until analysis. The plasma concentration of Fab’_BCMA_‐MORF1 was measured using fluorescence detection at the IRDye 750 wavelength (excitation/emission: 750/780 nm) with a Tecan plate reader. Standard curves were generated using known concentrations of IRDye 750‐labeled Fab’_BCMA_‐MORF1 to quantify the plasma levels at each time point. Half‐life (t_1/2_), area under the concentration‐time curve (AUC), clearance (CL), and volume of distribution (Vd), were calculated using PKsolver based on a two‐compartmental model. Results were reported as mean ± standard deviation (SD) for each time point.

### In Vivo Toxicity Study

The in vivo cytotoxicity of the treatment was assessed using NRG mice (n = 3). The mice were inoculated intravenously with MM.1S cells at a dose of 3 × 10^6^. Human peripheral blood mononuclear cells (PBMCs) were injected intravenously into each mouse at a dose of 3 × 10^7^ cells per mouse to establish a human immune system context at day 3. After 4 h, different treatments: BCMA MATCH and Teclistamab was injected to the mice. Twenty‐four hours post‐treatment, mice were euthanized, and samples were collected from the spleen, peripheral blood, and bone marrow. Spleen and bone marrow cells were isolated by mechanical dissociation, followed by red blood cell lysis. Peripheral blood was collected through cardiac puncture and processed to isolate mononuclear cells. All collected samples were analyzed for the presence of MM.1S cells and human PBMCs to assess cytotoxic activity. Flow cytometry was used to quantify remaining MM.1S cells in each tissue, using specific markers for MM.1S and human immune cells, enabling assessment of immune‐mediated cytotoxicity. The reduction in MM.1S cell populations in the spleen, peripheral blood, and bone marrow was compared to controls, providing a measure of the in vivo efficacy of PBMC‐mediated killing of MM.1S cells in this model.

### Statistical Analysis

All data were analyzed using GraphPad Prism software. Results were presented as mean ± standard deviation (SD) from at least three independent experiments, unless otherwise specified. Multiple comparisons between groups were performed using one‐way or two‐way analysis of variance (ANOVA) followed by Tukey post hoc test. For comparisons between two groups, an unpaired two‐tailed Student's t‐test was used. Significance levels were indicated as follows: ns (not significant), *p < 0.05, **p < 0.01, ***p < 0.001, and ****p < 0.0001. Graphs were generated using GraphPad Prism.

## Conflict of Interest

The authors declare the following competing financial interest: JY and JK are co‐inventors on a pending US patent application related to this work; it is assigned to the University of Utah.

## Supporting information



Supporting Information

## Data Availability

The data that support the findings of this study are available from the corresponding author upon reasonable request.
