# Supplementary material for: Self‐Assembling Multi‐Antigen T Cell Hybridizers for Precision Immunotherapy of Multiple Myeloma
Source: Adv Healthc Mater. 2025 Aug 1;14(29):e02156. doi: 10.1002/adhm.202502156 (PMC12616583; doi:10.1002/adhm.202502156)
Supplement: Supplementary file 1 — Supporting Information [file ADHM-14-0-s001.pdf]

ADVANCED HEALTHCARE MATERIALS

Supporting Information

for *Adv. Healthcare Mater.*, DOI 10.1002/adhm.202502156

Self-Assembling Multi-Antigen T Cell Hybridizers for Precision Immunotherapy of Multiple Myeloma

Shannuo Li, Jiahui Li, Hasan Al Faruque, Paul Shami, Birgit Knoechel, Jens Lohr, Douglas Sborov, Jiyuan Yang and Jindřich Kopeček**

Supporting Information

Self-Assembling Multi-Antigen T Cell Hybridizers for Precision Immunotherapy of Multiple Myeloma

Shannuo Li[1,2], Jiahui Li[1,2], Hasan Al Faruque[1,2], Paul Shami[3], Birgit Knoechel[3], Jens Lohr[3], Douglas Sborov[3], Jiyuan Yang[1,2], Jindřich Kopeček[1,2,4*]*

S. Li, J. Li, H. Faruque, J. Yang, J. Kopeček
[1]Department of Molecular Pharmaceutics,
[2]Center for Controlled Chemical Delivery, University of Utah, Salt Lake City, Utah 84112, USA
E-mail: jindrich.kopecek@utah.edu, jiyuan.yang@utah.edu

P. Shami, B. Knoechel, J. Lohr, D. Sborov
[3]Huntsman Cancer Institute, University of Utah, Salt Lake City, UT 84112, USA

J. Kopeček
[4]Department of Biomedical Engineering, University of Utah, Salt Lake City, Utah 84112, USA

Contents

Materials and Methods

Figure S1. Size-exclusion chromatography (SEC) profiles of Fab'$_{CD3}$-MORF2, Fab'$_{SLAMF7}$-MORF1, and their hybridized complex.
Figure S2. Hydrodynamic diameters of Fab'-MORF conjugates measured by dynamic light scattering (DLS).
Figure S3. UV-Vis spectroscopy showing hypochromic effect upon hybridization of complementary MORFs.
Figure S4. Thermal and structural stability of Fab'-MORF conjugates assessed by SEC and CD spectroscopy.
Figure S5. Biorecognition and cell surface hybridization of Fab'$_{BCMA}$-MORF1 and Fab'$_{CD3}$-MORF2 visualized by confocal microscopy.
Figure S6. Flow cytometric analysis of T cell activation and degranulation
Figure S7. Assessment of T cell activation markers and co-stimulatory molecule expression on MM cells.
Figure S8. Cultured T cells maintain cytotoxic functionality comparable to freshly isolated T cells.
Figure S9 Separate titration experiments were conducted using Fab'$_{BCMA}$-MORF1 and Fab'$_{CD3}$-MORF2
Figure S10. Fas (CD95) expression upregulated following co-culture MATCH treatment.
Figure S11. Control studies of *in vitro* apoptosis by Annexin V/PI binding assay.
Figure S12. Flow cytometric gating strategy for identifying major immune cell subsets in patient-derived samples.
Figure S13. Representative microscopy images of patient-derived bone marrow mononuclear cells (BM-MNCs) following 48 h treatments.
Figure S14. Patient sample responses to the BCMA or CD38 MATCH treatment versus the macrophage percentage in immune composition.
Figure S15. Pharmacokinetics and biodistribution of Fab'$_{BCMA}$-MORF1-IRdye750

Materials and Methods

Detection of hybridization by UV-visible spectroscopy

Hybridization between Fab'-MORF1 and Fab'-MORF2 was assessed by analyzing the hypochromic effect using a UV-vis spectrophotometry (Nanodrop, ND-1000 Spectrophotometer). Fab'-MORF1 and Fab'-MORF2 were each dissolved in PBS (pH 7.4) at a concentration of 2.5 μM (MORF equivalent) and mixed at varying molar ratios while keeping the total MORF concentration constant at 2.5 μM. For example, an 80:20 Fab'-MORF1 to Fab'-MORF2 mixture was prepared by combining 0.8 mL of Fab'-MORF1 (2.5 μM) with 0.2 mL of Fab'-MORF2 (2.5 μM). The absorbance at 260 nm was measured to detect hybridization-induced hypochromicity, indicative of base stacking and duplex formation. All measurements were performed in triplicate to ensure reproducibility.

Dynamic light scattering (DLS)

The hydrodynamic diameters of Fab'-MORF1 and Fab'-MORF2, along with their respective precursors (whole antibody and $F(ab')_2$), were characterized by dynamic light scattering (DLS, Malvern). Measurements were performed at room temperature in PBS (pH 7.4). Samples were filtered through a 0.22 μm syringe filter prior to analysis.

Circular dichroism (CD) spectrometry

CD spectra were acquired using an Aviv 62DS CD spectrometer equipped with thermoelectric temperature control (Aviv Biomedical, Lakewood, NJ). Standard spectral scans (excluding thermal melting) were conducted at 25 °C, recording from 300 to 200 nm at 1 nm intervals (bandwidth = 1 nm, 2 sec per step). Samples were prepared in PBS (pH 7.4) at 50 μM MORF-equivalent concentrations. All samples were filtered through a 0.22 μm membrane and loaded into a 0.1-cm path length quartz cuvette. Background spectra (PBS only) were subtracted from each sample spectrum, and data from three consecutive scans were averaged. For thermal melting analysis, Fab'-MORF1 and Fab'-MORF2 (or MORF1 and MORF2) were mixed in an equimolar ratio (5 μM each, based on MORF equivalents) and incubated for 1 h at room temperature in PBS (pH 7.4). After filtration, the mixture was transferred to a 1-cm path length quartz cuvette. A forward temperature scan was first performed from 25 °C to 95 °C in 2 °C increments, allowing 2 min equilibration followed by 30 sec data acquisition at each step. A reverse scan followed, cooling from 95 °C to 25 °C in 10 °C decrements, with 5 min equilibration and 30 sec data acquisition per step.

CD signal at 260 nm was monitored (n = 3) and observed ellipticity (θ_{obs}) was converted to molar ellipticity (θ) using the formula: $\theta = \theta_{obs} / (l \times c)$, where *l* is the optical path length in cm, and *c* is the molar concentration (MORF equivalent). To determine the melting temperature (T_m) of MORF1-MORF2 hybridization, θ (at 260 nm) was plotted as a function of temperature (T) and fitted to a four-parameter logistic model using GraphPad Prism.

PK study of Fab'$_{BCMA}$-MORF1

The pharmacokinetics of Fab'$_{BCMA}$-MORF1 was evaluated in NRG mice using an IRDye 750-labeled version of the molecule for fluorescent tracking. Tumor-bearing and non-tumor bearing NRG mice (n=3) were administered a single intravenous dose of IRDye 750-labeled Fab'$_{BCMA}$-MORF. Blood samples were collected at predetermined time points post-administration (0.17 h, 0.5 h, 1 h, 1.5 h, 2 h, 4 h, 6 h, 8 h, and 24 h) via tail vein bleeding. Plasma was separated by centrifugation and stored at 4°C until analysis. The plasma concentration of Fab'$_{BCMA}$-MORF1 was measured using fluorescence detection at the IRDye 750 wavelength (excitation/emission: 750/780 nm) with a Tecan plate reader. Standard curves were generated using known concentrations of IRDye 750-labeled Fab'$_{BCMA}$-MORF1 to

quantify the plasma levels at each time point. Half-life ($t_{1/2}$), area under the concentration-time curve (AUC), clearance (CL), and volume of distribution (Vd), were calculated using PKsolver based on a two-compartmental model. Results are reported as mean ± standard deviation (SD) for each time point.

In vivo toxicity study

The in vivo cytotoxicity of the treatment was assessed using NRG mice (n = 3). The mice were inoculated intravenously with MM.1S cells at a dose of 3 x 10^6. Human peripheral blood mononuclear cells (PBMCs) were injected intravenously into each mouse at a dose of 3 x 10^7 cells per mouse to establish a human immune system context at day 3. After 4 h, different treatments: BCMA MATCH and Teclistamab was injected to the mice.
Twenty-four hours post-treatment, mice were euthanized, and samples were collected from the spleen, peripheral blood, and bone marrow. Spleen and bone marrow cells were isolated by mechanical dissociation, followed by red blood cell lysis. Peripheral blood was collected through cardiac puncture and processed to isolate mononuclear cells. All collected samples were analyzed for the presence of MM.1S cells and human PBMCs to assess cytotoxic activity.

Flow cytometry was used to quantify remaining MM.1S cells in each tissue, using specific markers for MM.1S and human immune cells, enabling assessment of immune-mediated cytotoxicity. The reduction in MM.1S cell populations in the spleen, peripheral blood, and bone marrow was compared to controls, providing a measure of the in vivo efficacy of PBMC-mediated killing of MM.1S cells in this model.

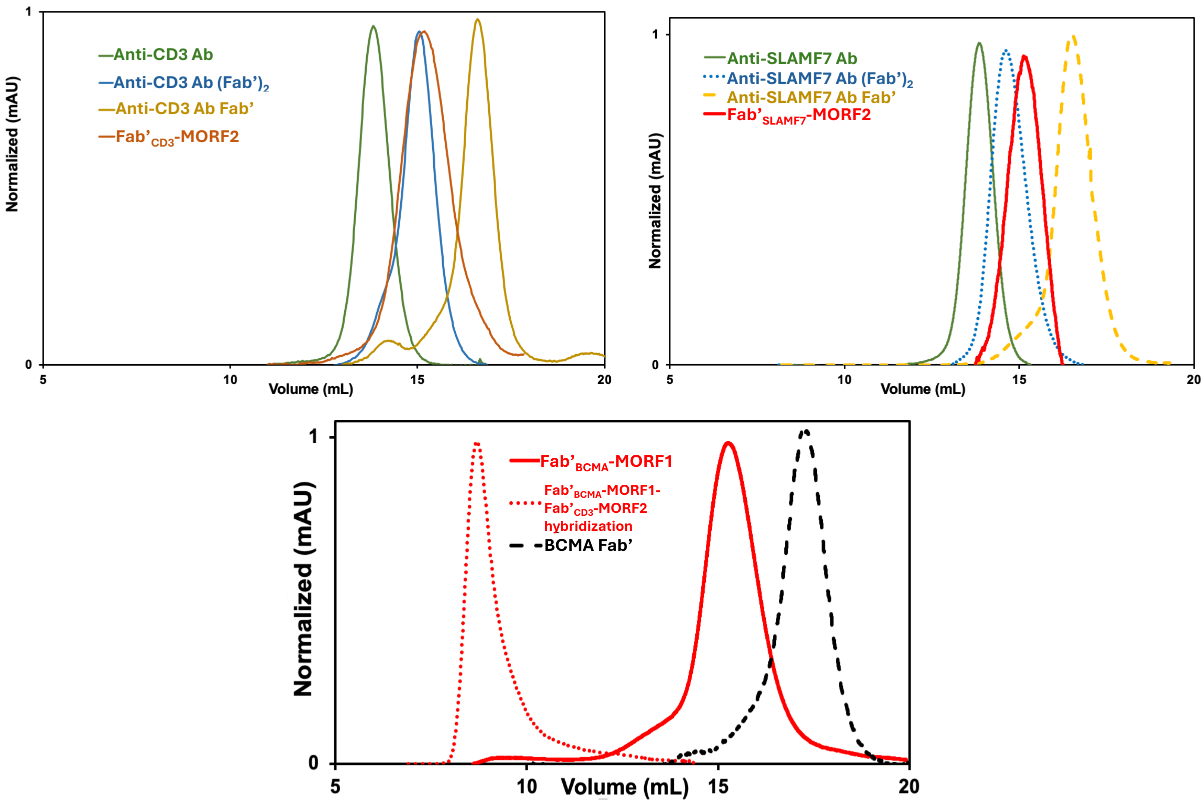

Figure S1. Size-exclusion chromatography (SEC) profiles of Fab'$_{CD3}$-MORF2, Fab'$_{SLAMF7}$-MORF1, and Fab'-MORFs hybridized complex, analyzed using a Superdex 200 column. Shifts in elution volumes confirm successful conjugation of MORF oligonucleotides to Fab' fragments and the formation of the hybridized complex through MORF1–MORF2 pairing.

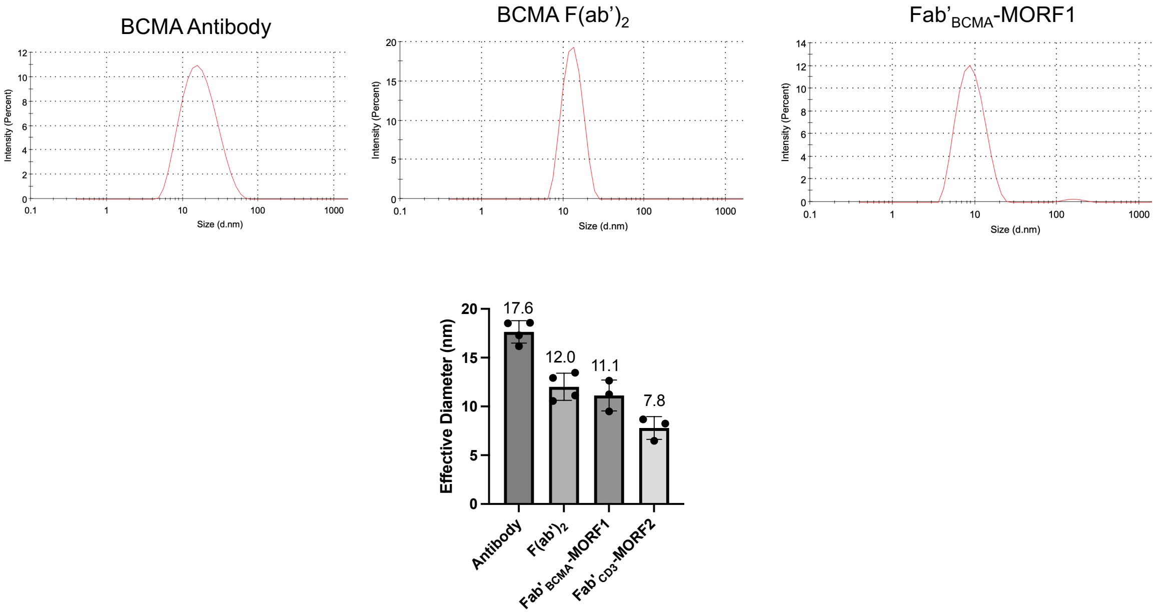

Figure S2. Hydrodynamic diameters of the whole antibody, F(ab')2, Fab'$_{\mathrm{BCMA}}$-MORF1, and Fab'$_{\mathrm{CD3}}$-MORF2, as measured by dynamic light scattering (DLS). All samples were prepared in PBS (pH 7.4). Data are presented as mean ± SD.

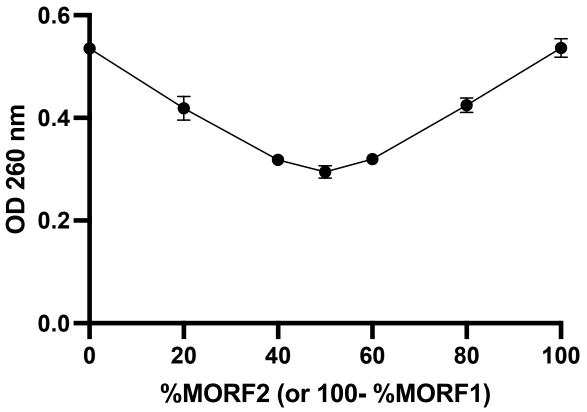

Figure S3. Hypochromic effect upon hybridization of Fab'$_{\mathrm{BCMA}}$-MORF1 and Fab'$_{\mathrm{CD3}}$-MORF2, analyzed by UV-Vis spectrophotometry. A decrease in optical density (OD) at 260 nm was observed as the two MORF conjugates were mixed in varying ratios in PBS (pH 7.4), indicating duplex formation. The most pronounced hypochromic shift occurred at a 1:1 ratio, consistent with optimal hybridization. Data are presented as mean ± SD (n = 3).

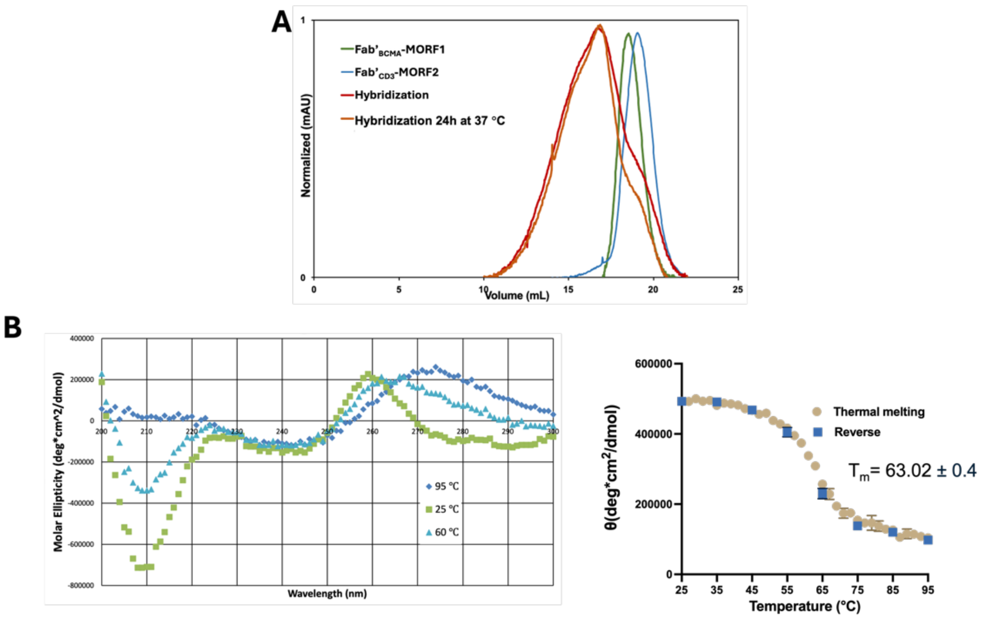

Figure S4. (A) Stability of Fab'-MORF conjugates. SEC analysis using an S6a column demonstrated that Fab'$_{BCMA}$-MORF1 and Fab'$_{CD3}$-MORF2 maintained hybrid stability after 24 h incubation at 37 °C (orange curve). (B) Thermal stability of MORF1–MORF2 hybrids assessed by circular dichroism (CD) spectroscopy. CD spectra of equimolar MORF1 and MORF2 (5 µM each in PBS, pH 7.4) were recorded at 25 °C, 60 °C, and 95 °C. Upon heating, the characteristic positive band at 260 nm shifted toward 275 nm, indicating conformational changes during thermal denaturation. Molar ellipticity (θ) at 260 nm was tracked to generate a thermal melting curve, which exhibited a sigmoidal decrease with increasing temperature. Fitting to a logistic function yielded a melting temperature (T_m) of 63.0 °C. Comparable results were obtained during the cooling phase, confirming the thermal reversibility of MORF1–MORF2 hybridization. Data are presented as mean ± SD (n = 3).

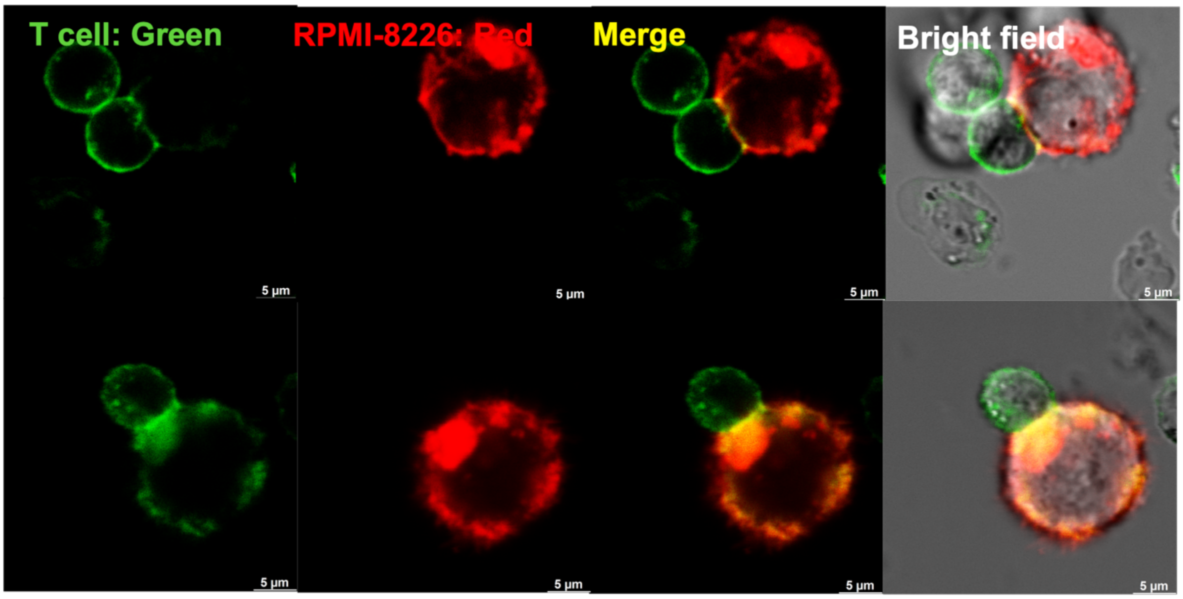

Figure S5. Biorecognition and cell surface hybridization of Fab’$_{BCMA}$-MORF1 and Fab’$_{CD3}$-MORF2. Confocal microscopy images show co-localization of T cells (green, stained with CellMask™ Green; Thermo Fisher) and RPMI-8226-Lck-mScarlet cells following treatment with a 50 µM equivalent dose of premixed BCMA-based MATCH. The appearance of yellow fluorescence at the interface between cells indicates immune synapse formation and successful hybridization of complementary MORFs. Scale bars are provided in the figure panels.

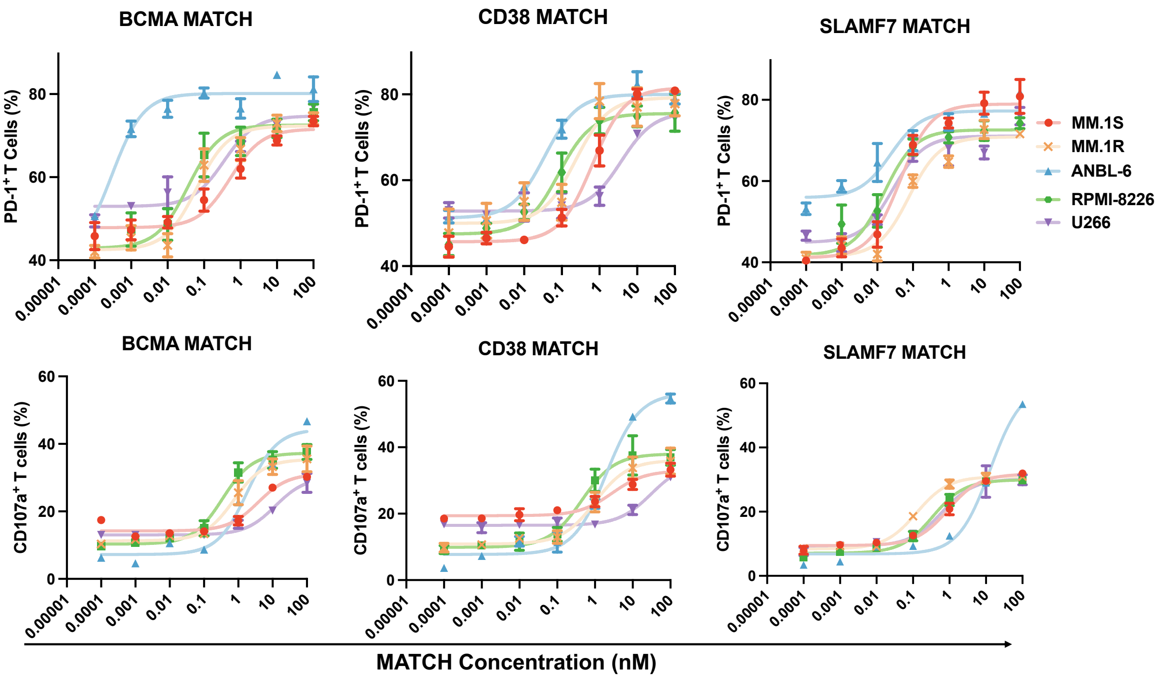

Figure S6. Flow cytometric analysis of T cell activation and degranulation in response to increasing concentrations of BCMA-targeted MATCH from 0.0001 nM to 100 nM. Human primary T cells were co-cultured with MM.1S cells in the presence of titrated amounts of premixed Fab'$_{BCMA}$-MORF1 and Fab'$_{CD3}$-MORF2. The proportion of PD-1^{+} T cells (a marker of activation/exhaustion) and CD107a^{+} T cells (a marker of degranulation) was quantified after 24 h. A concentration-dependent increase in both PD-1 and CD107a expression was observed. Data are representative of at least three independent experiments.

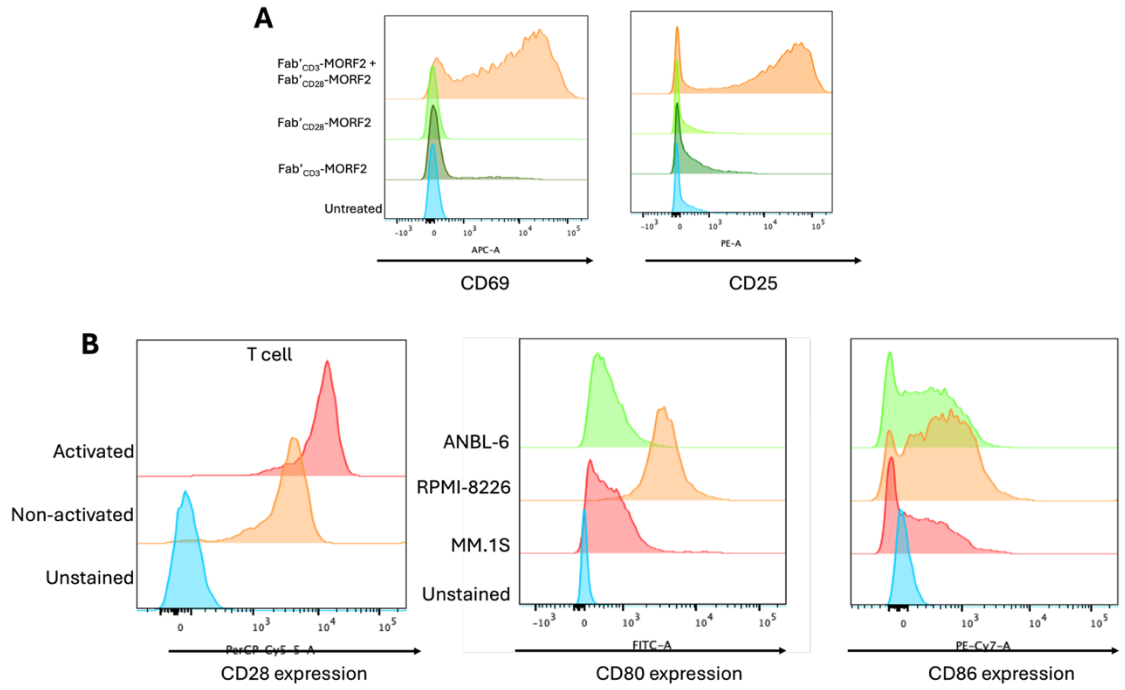

Figure S7. Assessment of T cell activation markers and co-stimulatory molecule expression on MM cells. (A) Expression of early (CD69) and intermediate (CD25) activation markers on human primary T cells following exposure to MATCH components, as measured by flow cytometry. (B) Surface expression of co-stimulatory ligands CD80 and CD86 on MM cell lines, which may contribute to T cell activation via CD28 signaling. Data support the presence of accessory signals that could lower the threshold for T cell activation in the MATCH system.

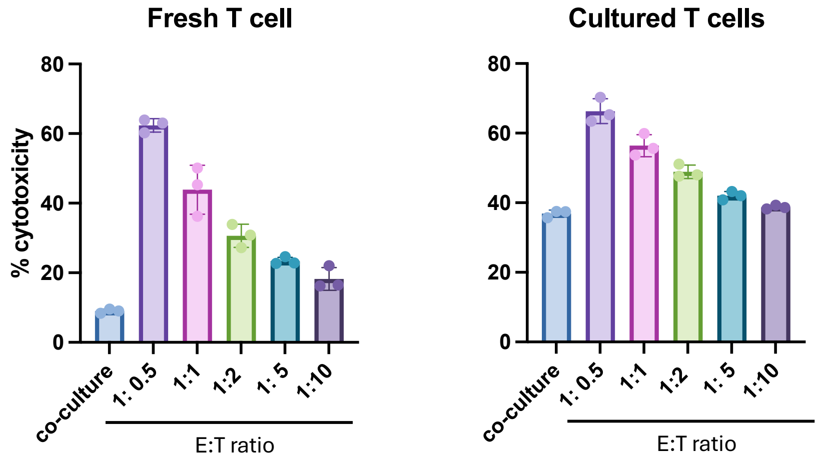

Figure S8. Cultured T cells maintain cytotoxic functionality comparable to freshly isolated T cells. MATCH-mediated cytotoxicity assays were performed using both freshly isolated, or short-term cultured human T cells. Results demonstrate that cultured T cells retain their ability to effectively kill multiple myeloma cells, indicating preserved effector function following in vitro expansion and stimulation. However, T cells are more readily activated when they co-culture with tumor cells after pre-activation in cell culture, which lowers the threshold for T cell activation.

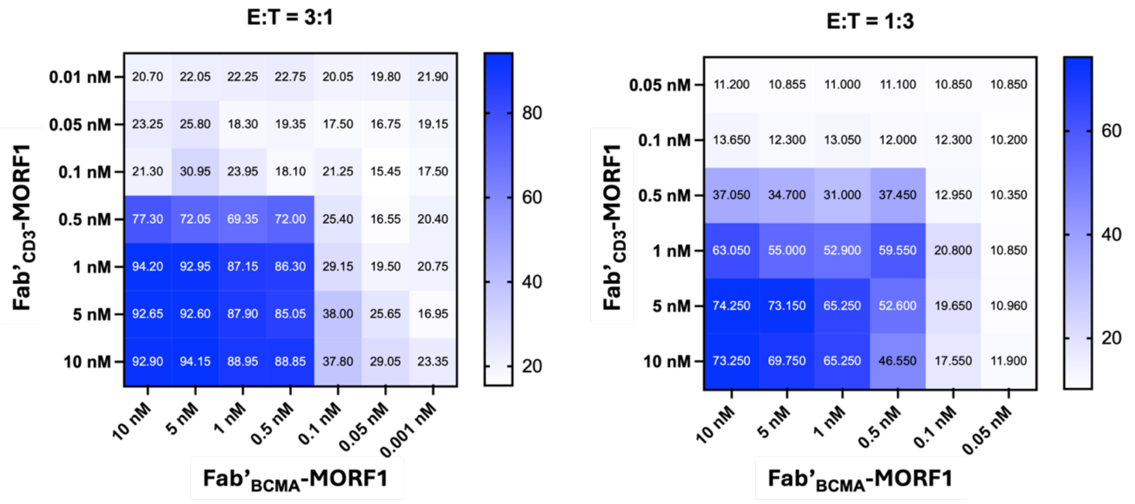

Figure S9. Separate titration experiments were conducted using Fab'$_{BCMA}$-MORF1 and Fab'$_{CD3}$-MORF2 on MM.1S cells at two different effector-to-target (E:T) ratios—1:3 and 3:1—representing T cell-limited and tumor cell-limited conditions, respectively. A more pronounced reduction in cytotoxic activity was observed with decreasing concentrations of Fab'$_{CD3}$-MORF2, highlighting the essential role of CD3 engagement in driving T cell activation and tumor cell killing.

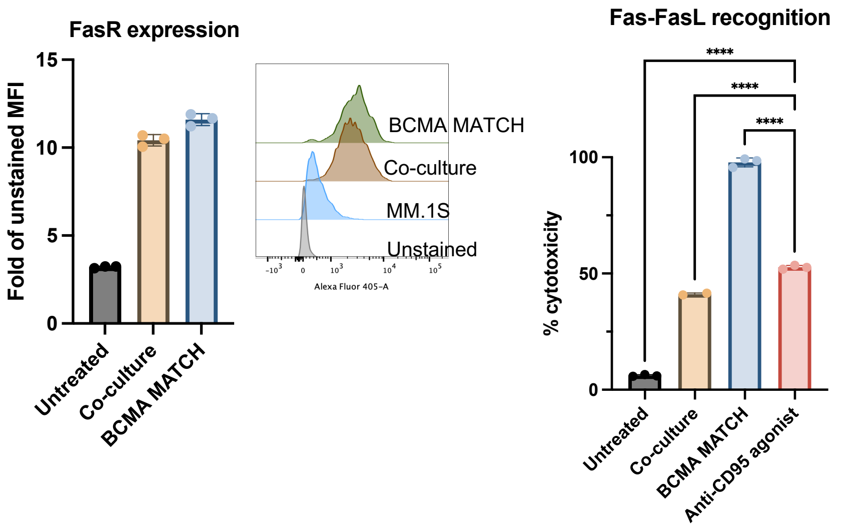

Figure S10. Fas (CD95) expression upregulated following co-culture MATCH treatment. Flow cytometry analysis shows increased Fas expression on MM.1S cells after treatment with BCMA-targeted MATCH. To evaluate the functional relevance of Fas signaling, MM.1S cells were treated with an agonistic anti-Fas antibody (CH-11). Cell death induced by CH-11 was compared to that observed in T cell co-culture alone or with BCMA MATCH treatment.

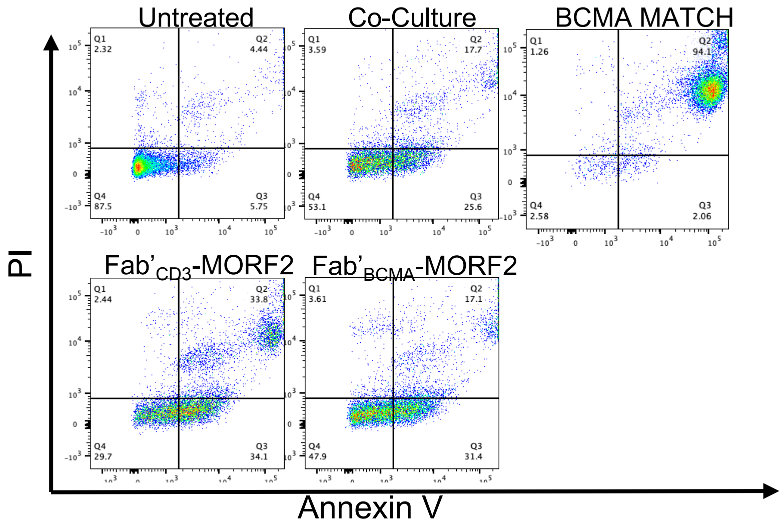

Figure S11. Control studies of *in vitro* apoptosis by Annexin V/PI binding assay. Apoptosis induction of MM.1S. Incubation time was 24 h. The following indications apply: Untreated: in cell medium without IL-2; Co-Culture: T cells and MM.1S cells in cell medium without IL-2; BCMA MATCH; 50 nM of Fab'$_{BCMA}$-MORF1 and Fab'$_{CD3}$-MORF2 in equivalent of MORF1-MORF2 concentration. Fab'$_{CD3}$-MORF2: only add 50 nM Fab'$_{CD3}$-MORF2; Fab'$_{BCMA}$-MORF1: only add 50 nM of Fab'$_{BCMA}$-MORF1.

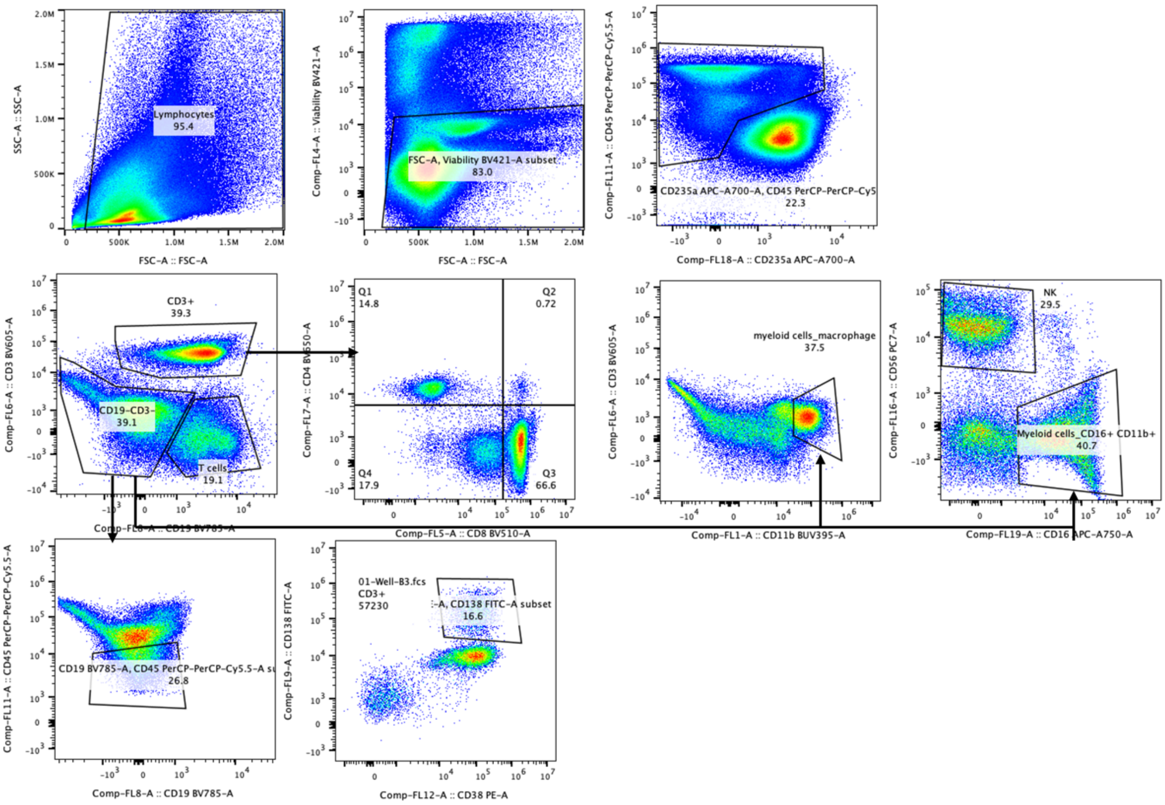

Figure S12. **Flow cytometric gating strategy for identifying major immune cell subsets in patient-derived samples. Cells** were first gated on forward and side scatter (FSC/SSC) to isolate the lymphocyte population. A viability dye (DAPI) was used to exclude nonviable cells. Cells negative for CD235a (an erythrocyte marker) were further gated. Within the $CD45^+$ population, T cells ($CD3^+$), and B cells ($CD19^+$) were further gated. A quadrant plot was used to differentiate T cell subpopulations or further define surface markers CD4 and CD8. Myeloid-lineage cells ($CD45^+$, SSC^{high}, $CD11b^+$) were gated out. Another plot distinguished NK cells using CD56. The percentage of multiple myeloma cells ($CD138^+CD38^+CD19^-CD45^-$) were calculated from the total non-RBC population.

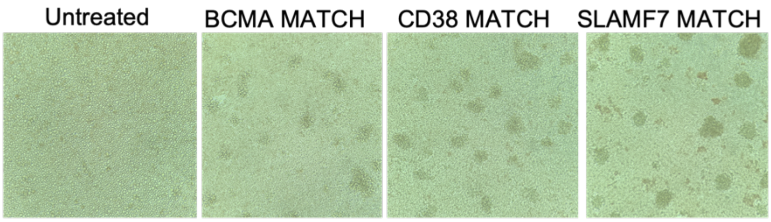

Figure S13. Representative microscopy images of patient-derived bone marrow mononuclear cells (BM-MNCs) following 48 h treatments.

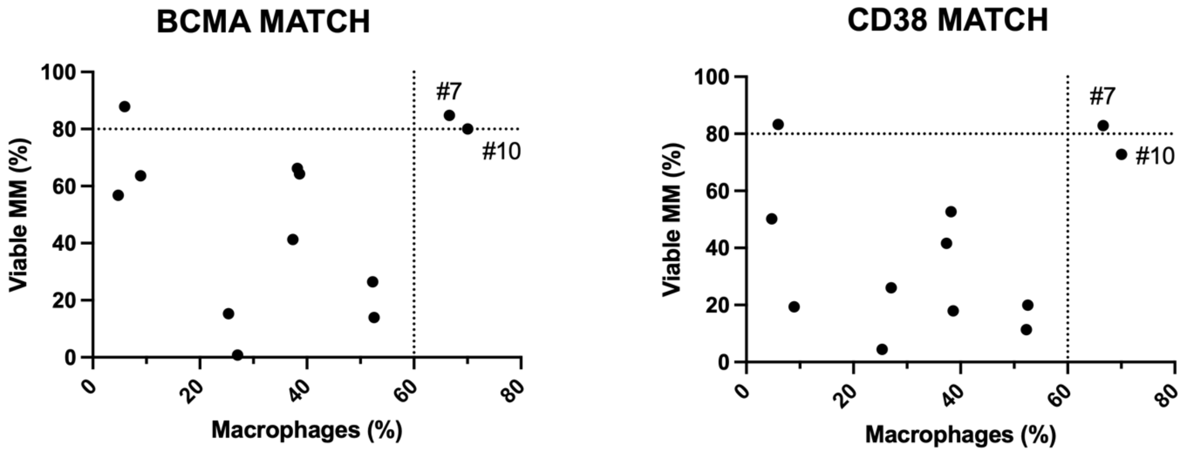

Figure S14. Patient sample responses to the BCMA or CD38 MATCH treatment versus the macrophage percentage in immune composition.

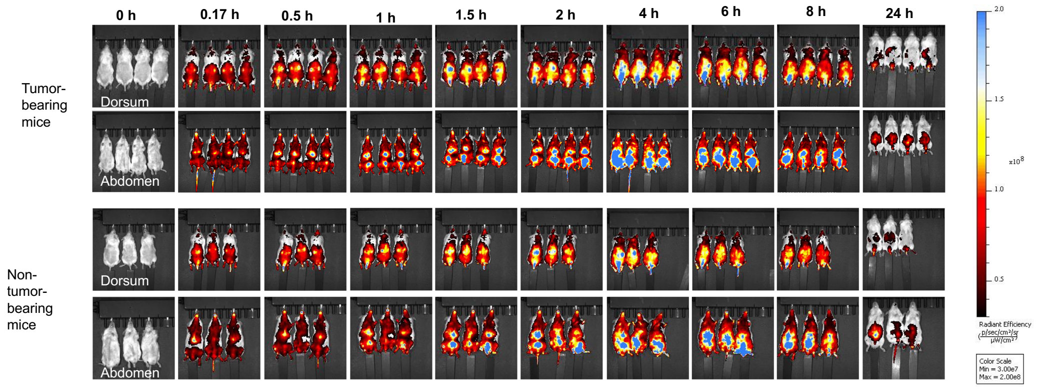

Figure S15. IVIS-based biodistribution analysis of Fab'$_{BCMA}$-MORF1-IRdye 750 from injection up to 24 hours post-administration. Fluorescence signals showed initial accumulation in major organs within 2–8 hours, followed by a marked decline by 24 hours, indicating efficient systemic clearance and minimal long-term tissue retention.
